# Bridge health monitoring using existing telecommunication fiber-optic networks

**DOI:** 10.1038/s41467-026-76935-0

**Published:** 2026-08-21

**Authors:** Jingxiao Liu, Jatin Aggarwal, Doyun Hwang, Jigu Lee, Yuyan Wu, Fu Yin, Haipeng Li, Paolo Santi, Hoon Sohn, Biondo Biondi, Carlo Ratti, Hae Young Noh

**Affiliations:** 1 Senseable City Laboratory, Massachusetts Institute of Technology, Cambridge, MA USA; 2 School of Civil and Environmental Engineering, Nanyang Technological University, Singapore, Singapore; 3 Department of Civil and Environmental Engineering, Stanford University, Stanford, CA USA; 4 Department of Civil and Environmental Engineering, Korea Advanced Institute of Science and Technology, Daejeon, Republic of Korea; 5 Department of Earth, Environmental, and Planetary Sciences, Rice University, Houston, TX USA; 6 Department of Geophysics, Stanford University, Stanford, CA USA; 7 Istituto di Informatica e Telematica del CNR, Pisa, Italy; 8 Dipartimento ABC, Politecnico di Milano, Milan, Italy

**Keywords:** Civil engineering, Mechanical engineering, Electrical and electronic engineering

## Abstract

Aging bridges pose significant risks to public safety. Although existing sensing technologies offer a potential solution for structural health monitoring, the high cost and complexity of installing and maintaining sensing systems have limited their scalability and long-term deployment. Here, we present a scalable and cost-effective approach that turns pre-existing telecommunication (telecom) fiber-optic cables into a dense array of dynamic strain sensors to continuously monitor bridges. Using telecom fibers, we retrieve guided wavefields across bridge superstructures, which was impossible with existing sensing technologies due to their limited scalability and the prohibitive cost of ultra-dense sensor arrays required to capture meter-scale wavelengths. We also estimate quasi-static displacements with millimeter-level accuracy and extract modal parameters. These results demonstrate that our approach is robust to noise from imperfect fiber-bridge coupling, a limitation inherent to the non-dedicated nature of telecom fibers used as sensors. Furthermore, we demonstrated the effectiveness of this approach through field evaluations across six bridges in the United States and South Korea. Because telecom fibers are widely deployed and co-located with civil infrastructure systems, this study introduces a new sensing paradigm that leverages existing fiber-optic networks to enable large-scale, real-time structural monitoring without the cost and logistical burden of dedicated sensors.

## Abstract (web summary)

This study demonstrates that existing telecommunication fiber-optic cables can be used as dense, distributed strain sensors for structural health monitoring of bridges, enabling meter-scale measurements of guided wavefields, displacements, and modal properties across multiple bridge structures.

## Introduction

Modern societies face growing challenges from aging infrastructure, driven by both physical deterioration and persistent gaps in structural response data and condition assessment. These information deficits increase the risk of catastrophic failures and unexpected service disruptions, while exacerbating the widening infrastructure funding gap. Bridges, in particular, highlight the urgency of the issue. According to the latest ASCE Infrastructure Report Card^1^, 6.8% of the 623,218 bridges in the United States—more than 42,300 in total—are in poor condition and require replacement or major rehabilitation. By 2025, their average age will reach 47 years, nearing the 50-year design life of many bridges and placing a significant portion of the network at risk of further degradation. Aging, rising traffic volumes, and environmental stressors compound these vulnerabilities. To mitigate these risks, structural health monitoring (SHM) is essential for early damage detection, enabling informed maintenance decisions, extending service life, and improving public safety^2,3^.

SHM techniques have made significant strides in assessing the physical condition of infrastructure. On-site inspection techniques, such as infrared thermography^4^, ultrasonic inspection^5^, and ground-penetrating radar^6^ are commonly used to detect material degradation and delamination. Developments in computer vision and robotics have enabled applications, such as automated crack detection and drone-based visual inspections^7^. In addition to visual and on-site inspection methods, wireless sensor networks using fixed instrumentation, such as accelerometers and strain gauges, allow for long-term tracking of structural responses and dynamic behavior^8,9^. More recently, mobile sensing strategies using vehicle-mounted accelerometers or smartphones have gained traction for bridge monitoring through analyzing vehicle–bridge interactions^10,11^, offering an alternative when fixed sensors are not feasible. Recent SHM and bridge-monitoring reviews highlight that, despite these advances, widespread field deployment remains limited by recurring bottlenecks, including sparse sensing, installation and maintenance cost, environmental and operational variability, and the difficulty of translating laboratory demonstrations into scalable in situ systems^12,13^. Farrar et al.^13^ describe these as stagnation points that have shaped the development of SHM over the last decades. They further note that guided-wave approaches, although highly sensitive to localized damage, have generally remained difficult to deploy for online, in situ monitoring. In bridge monitoring, these challenges are especially acute because resolving localized structural response requires dense spatial sensing, whereas conventional sensing systems remain costly to install and maintain over large infrastructure networks. As a result, most U.S. bridges are still inspected manually on a biennial basis, per the National Bridge Inspection Standards. The gap between available technology and routine practice underscores the pressing need for monitoring solutions that are scalable and cost-effective.

**Fig. 1:**
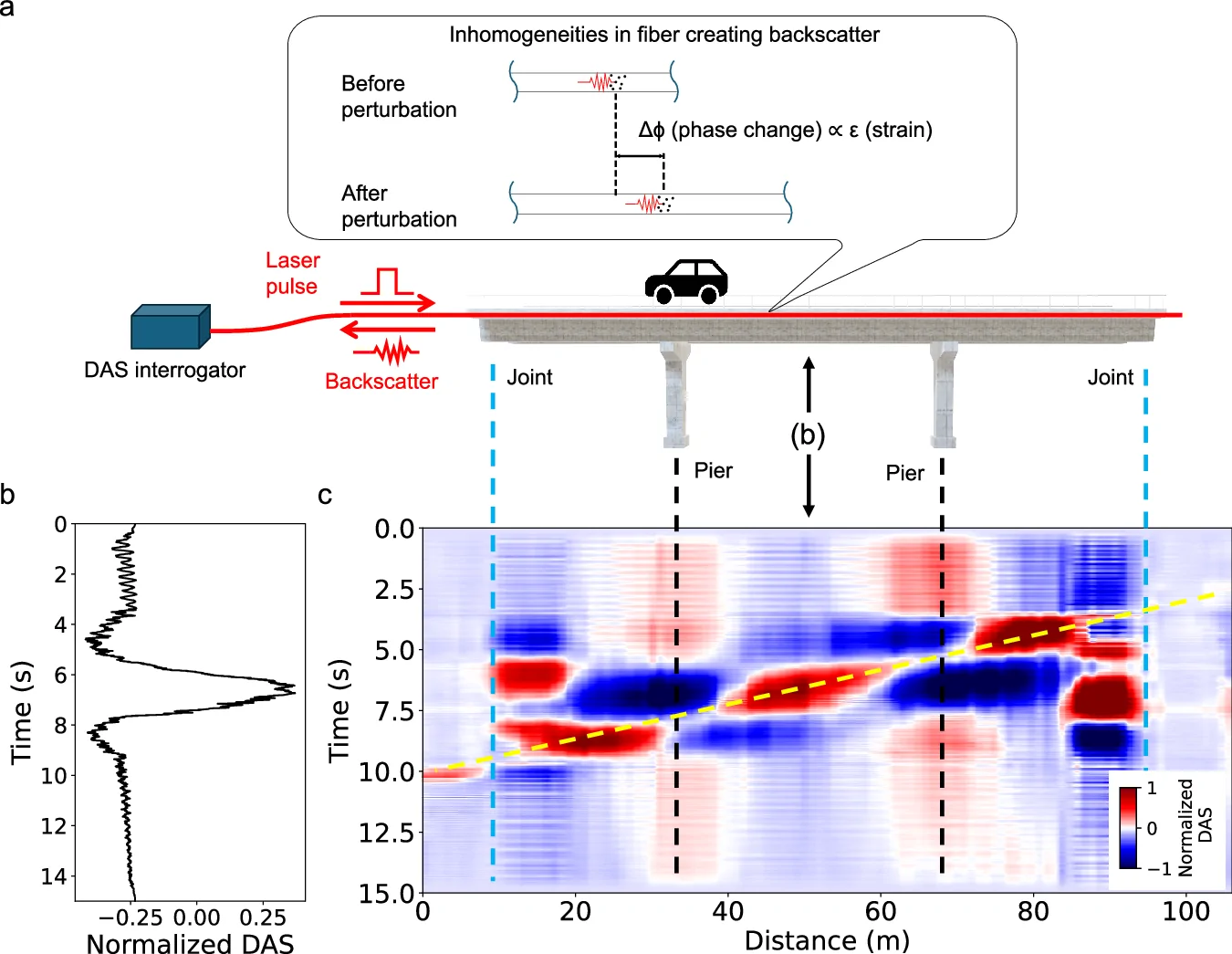
Monitoring bridge responses using Telecom as Sensors. **a** Schematic of the DAS measurement principle. Laser pulses are injected into a telecom fiber coupled to a three-span bridge. Microscopic inhomogeneities in the fiber cause Rayleigh backscattering. External strain, such as that induced by a passing vehicle, alters the optical path length, producing a phase shift (Δ*ϕ*) in the backscattered signal proportional to the axial strain *ϵ*. **b** Time series of the DAS response recorded at the mid-span (location marked by the double-headed arrow in (**a**, **c**), capturing vehicle-induced strain as the vehicle travels from right to left. Both dynamic and quasi-static components of the response are observed. **c** Space-time plot of normalized DAS response along the full bridge span. The vehicle’s passage from right to left produces clear spatiotemporal strain signatures. The yellow dashed line denotes the vehicle trajectory. Vertical dashed lines denote expansion joints and pier locations.

Here, we explore the use of pre-existing and ubiquitous urban infrastructure—telecom optical fibers—as a non-dedicated sensing platform to monitor bridge responses in a scalable and cost-effective way (Fig. 1). As of 2022, the U.S. had over 57 million miles of installed fiber-optic cables^14^, with more than half remaining “dark” (i.e., unused and reserved for future capacity). Because these fibers are often co-located with and mechanically coupled to civil infrastructure, such as bridges, we turn them into a dense array of dynamic strain sensors (hereafter referred to as “Telecom as Sensors”), transforming them into extensive sensing networks that require no additional maintenance beyond what is already provided for communication functions, redefining how and where structural monitoring can occur.

Telecom as Sensors is based on distributed acoustic sensing (DAS) technology. A typical DAS system consists of an interrogator and a standard optical fiber (Fig. 1a). The interrogator sends laser pulses into the fiber and measures the Rayleigh backscattered signal, which results from microscopic inhomogeneities in the fiber core^15^. By analyzing the phase shifts between the transmitted and backscattered signals, DAS retrieves axial dynamic strain or strain rate along tens to hundreds of kilometers of fiber with meter-scale resolution and kilohertz sampling frequency^16,17^. Furthermore, DAS measurements are time-synchronized and transmitted directly through the fiber. Compared to other sensors, such as cameras, accelerometers, and geophones, which are costly to deploy and offer limited spatial resolution, Telecom as Sensors provides meter-scale resolution and orders-of-magnitude greater coverage, at significantly lower marginal cost.

**Fig. 2:**
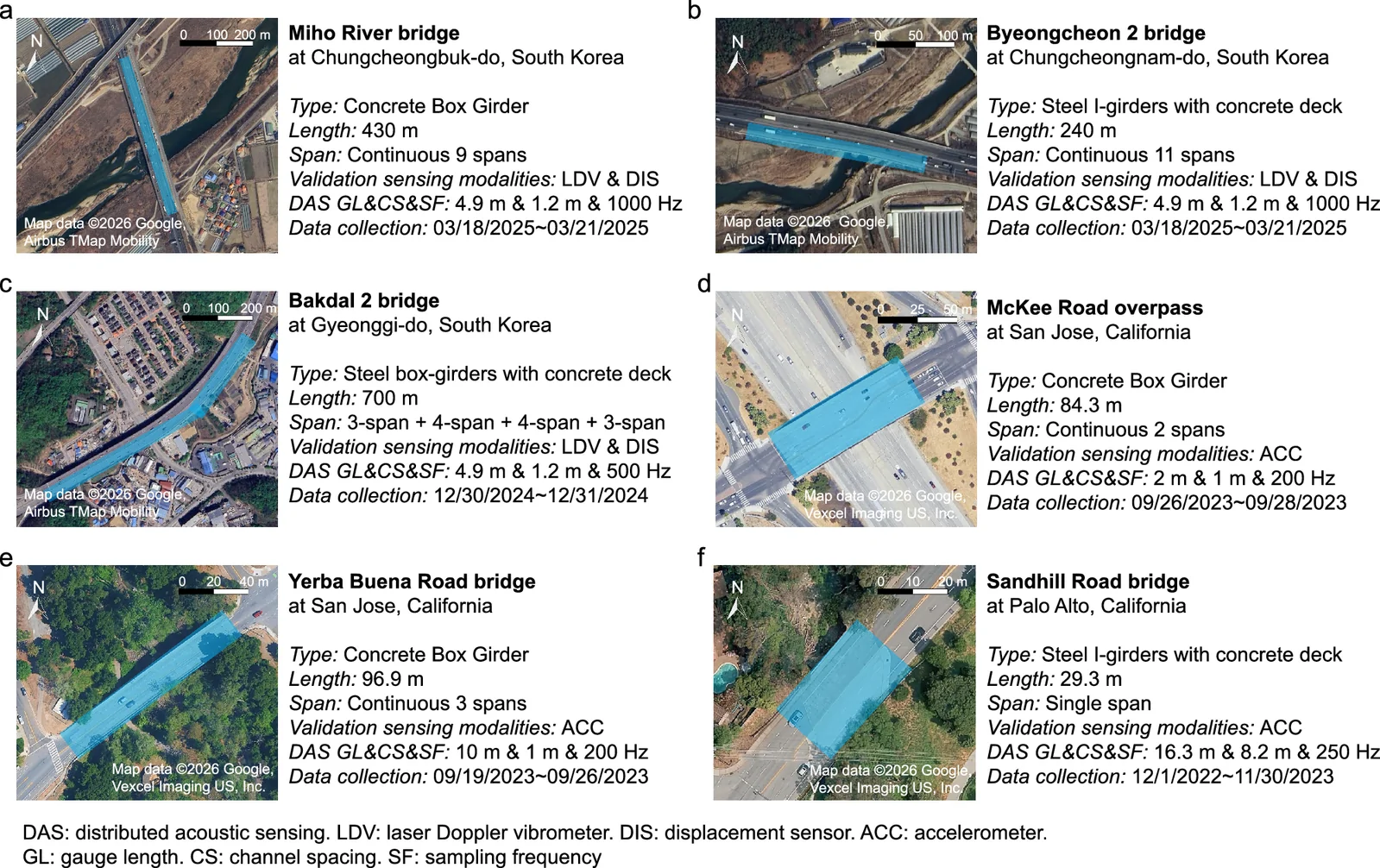
Aerial view (from Google Earth) and summary of six monitored bridges, detailing their structural type, span configuration, validation sensing modalities, DAS setup, and data collection period. The monitored bridge sections are highlighted in blue. The first three bridges (**a**–**c**) are located in South Korea, and the latter three (**d**–**f**) in California, USA.

Unlike using deployed optical fiber for sensing, Telecom as Sensors leverages existing telecom fibers that exhibit imperfect fiber-bridge coupling, a limitation inherent to their non-dedicated nature. Despite this, we demonstrate that Telecom as Sensors can accurately capture a comprehensive range of bridge responses, from propagating guided waves to quasi-static displacements and dynamic modal behavior, by integrating geophysical and structural monitoring approaches, including virtual source gathers and frequency domain decomposition (FDD) (see “Methods” for details), with the high spatial resolution of DAS. We show these capabilities on six bridges in the United States and South Korea, spanning diverse configurations and structural forms, such as concrete box girders and steel-concrete composite bridges (Fig. 2). Validation against conventional sensors and numerical models confirms the robustness and accuracy of the approach. Using Telecom as Sensors, we retrieve multi-mode guided wavefields across entire bridge superstructures, an achievement previously unattainable with existing sensing methods due to the prohibitive cost of ultra-dense sensor arrays required to capture meter-scale wavelengths. Our quasi-static displacement estimates achieve sub-millimeter accuracy (0.1 mm on average), and modal analyses yield an average of 87% agreement in mode shape identification compared to conventional measurements and numerical simulations. Additionally, we demonstrate a year-long monitoring of bridge dynamic responses, underscoring the method’s potential for continuous, long-term deployment.

Accurately capturing these structural responses provides critical insights into bridge condition and performance, which supports the detection of potential degradation or damage with high spatial and temporal resolution. Overall, this study establishes telecom fiber-optic networks as a scalable and cost-effective sensing platform for bridge monitoring, with broad implications for managing aging infrastructure and enhancing infrastructure resilience.

## Results

In this section, we present three main results from Telecom as Sensors for bridge monitoring: the analysis of guided wavefields, the estimation of quasi-static displacements, and the analysis of dynamic responses. A schematic overview of the analysis framework underlying these three results is provided in Supplementary Fig. 1.

### Analysis of multi-mode guided wavefields

**Fig. 3:**
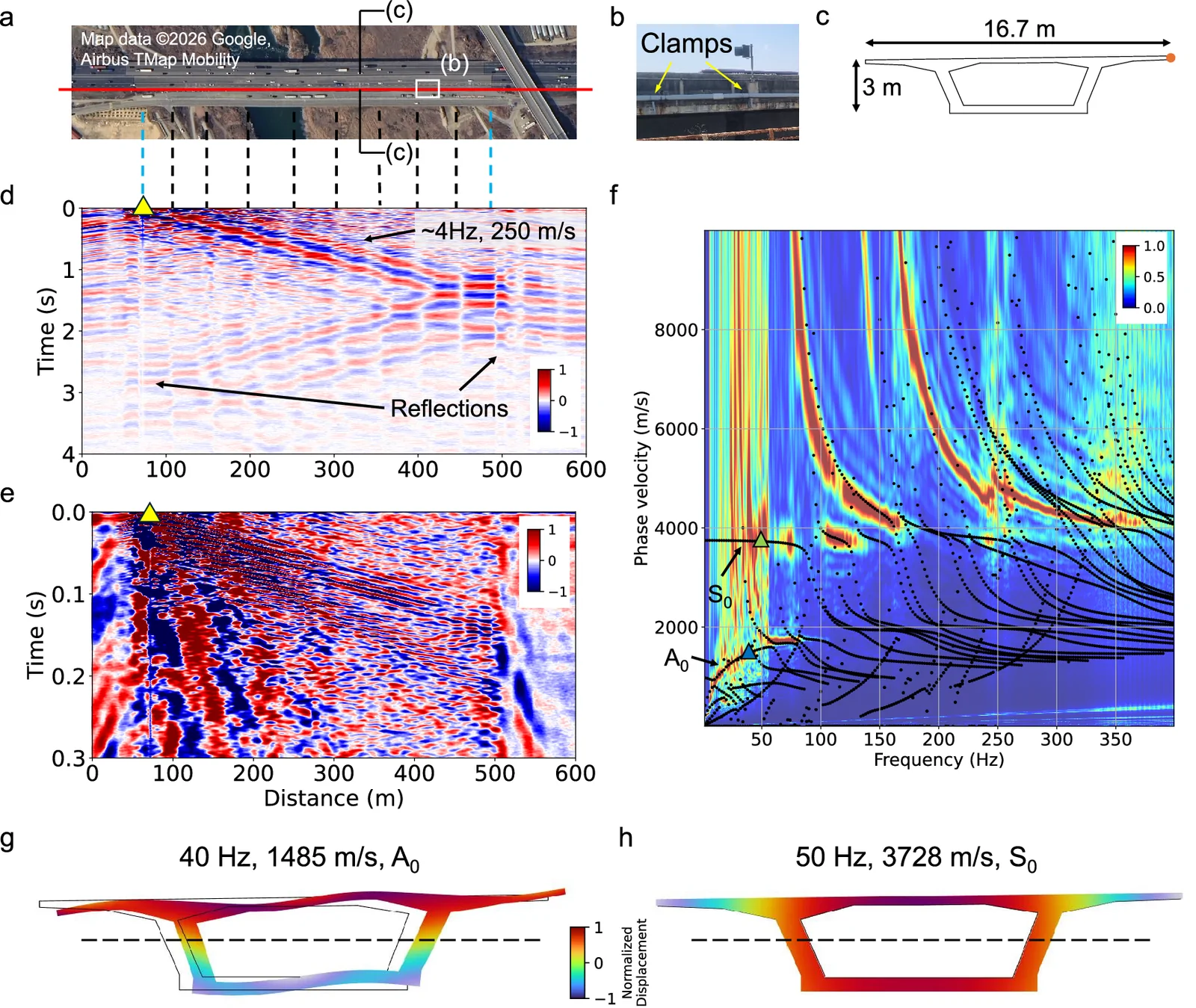
Analysis of guided wavefields in the Miho River Bridge using Telecom as Sensors. **a** Aerial view of the bridge from Google Earth, with the red line indicating the route of the telecom fiber-optic cable. Vertical dashed lines denote the locations of bridge piers (black) and expansion joints (blue). **b** Photo showing the side of the bridge where the telecom cable is mechanically clamped to the structure, with clamps spaced every 2.8 m. **c** Cross-section of the box girder at mid-span. The orange dot indicates the location of the mounted fiber. **d** Virtual source gather showing the propagation of guided waves along the bridge. The data are normalized for visualization. A 4 Hz wave propagating at approximately 250 m/s is visible and reflected at the expansion joints. The yellow triangle marks the virtual source location. **e** Zoomed-in view of (**d**) from 0 to 0.3 s, showing dispersive higher-frequency guided waves with faster velocities. **f** Dispersion image derived from the wavefield in (**d**), revealing multiple guided wave modes. The image is normalized by frequency to enhance visibility across the spectrum and is overlaid with dispersion curves obtained from SAFE-based guided wave modeling (black dots; see “Methods” for details). Simulation results show strong agreement with the observed dispersion patterns below 150 Hz. Blue and green triangles in (**f**) indicate the modes shown in (**g**, **h**), respectively. **g** Out-of-plane displacement of the *A*_0_ mode at 40 Hz (phase velocity 1485 m/s), antisymmetric about the central dashed line. **h** Out-of-plane displacement of the *S*_0_ mode at 50 Hz (phase velocity 3728 m/s), symmetric about the central dashed line.

The dense spatial resolution provided by Telecom as Sensors enables the retrieval of multi-mode guided wavefields in bridge superstructures, a capability unachievable with conventional SHM systems. Notably, this is achieved using existing telecom fibers, eliminating the need for dedicated sensor deployments. Unlike conventional vibration-based monitoring, which relies on sparse sensor networks and primarily captures global behavior through modal analysis, Telecom as Sensors offers orders of magnitude higher spatial resolution, yielding continuous wavefield measurements across the entire superstructure. These measurements are important, as variations in guided wave propagation characteristics are sensitive to changes in material properties, boundary conditions, and the presence of localized damage^18,19^. As an example, Fig. 3d, e show guided wavefields along the Miho River Bridge superstructure (Fig. 3a–c), derived using 4 days of DAS data. These results are obtained using ambient noise interferometry^20,21^ to retrieve the empirical Green’s function between station pairs, where the virtual source is set near the left expansion joint of the bridge (see “Methods” for details). The resulting wavefields reveal clear dispersive behavior of multiple guided wave modes. In Fig. 3d, a low-frequency content around 4 Hz propagates at approximately 250 m/s phase velocity, and its reflected phase is observed at the right expansion joint. Reduced wave amplitude is observed near the pier locations, due to changes in boundary conditions that scatter or dampen wave energy. In contrast, higher-frequency dispersive contents (waves above 100 Hz) attenuate more rapidly and display minimal amplitude loss at the piers (Fig. 3e). This behavior reflects their shorter wavelengths, which are less sensitive to changes in large-scale boundary conditions.

The dispersion image derived from the retrieved virtual shot gathers (VSGs) reveals at least five coherent guided wave modes, identifiable as red ridges in the dispersion spectrum, each exhibiting distinct dispersive characteristics (Fig. 3f). These dispersive guided waves can be regarded as Lamb wave-like modes^22^, which arise in plate-like structures of finite thickness and support multiple symmetric and antisymmetric modes. Although bridges are more complex than ideal plates, the deck and box girder components act as effective waveguides, and the retrieved dispersion curves show strong correspondence with Lamb-wave behavior. To validate these modes, we perform guided wave mode analysis using the semi-analytical finite element (SAFE) method^23^ (see “Methods” for details). The Miho River Bridge is a concrete box girder structure whose cross-sectional geometry is primarily the mid-span configuration (Fig. 3c), with modifications near the supports and end segments to enhance shear capacity. Since the mid-span geometry dominates the structure, the SAFE model simulates guided wave dispersion based on parameters extracted from the mid-span design drawings. The resulting dispersion curves are overlaid with the Telecom as Sensors-derived dispersion spectrum in Fig. 3f, showing strong agreement up to 150 Hz. In the figure, the agreement is evident where the red ridges of the measured modes align with the overlaid black theoretical curves, particularly for the fundamental symmetric (*S*_0_) and antisymmetric (*A*_0_) modes, as well as the first two higher-order modes. The *A*_0_ mode appears as the lowest-velocity branch in the dispersion image. It exhibits flexural motion, with the top and bottom surfaces of the bridge moving in opposite directions along the longitudinal axis—behavior consistent with the cross-sectional out-of-plane displacement field from the SAFE simulation (Fig. 3g). This mode in the dispersion spectrum originates at low frequencies (near 0 Hz) with a low phase velocity and increases steeply with frequency before gradually flattening beyond 80 Hz. The *S*_0_ mode shows relatively low dispersion at low frequencies (<100 Hz), with nearly constant phase velocities. This mode is characterized by longitudinal motion, where both surfaces move in the same direction, confirmed by the cross-sectional displacement field obtained from the SAFE simulation (Fig. 3h).

At frequencies higher than 75 Hz, additional wave modes appear above their respective cutoff frequencies. In this range, the observed dispersion curves result from a combination of responses from multiple cross-section types. The mid-span cross-section simulation agrees with the observed data up to about 150 Hz but does not capture a higher-order mode present between 175 and 350 Hz (Fig. 3f). However, the terminal segment cross-section provides a better match for this high-frequency mode (Supplementary Fig. 2). These differences highlight that although high-frequency, short-wavelength modes are less sensitive to large-scale boundary condition changes, they are more sensitive to local geometric and structural variations.

Propagating guided waves are also observed in the Byeongcheon 2 bridge, Bakdal 2 bridge, and McKee Road overpass (Supplementary Figs. 3–5). Due to variations in DAS system sampling rates, each site exhibits different upper frequency limits, further constrained by spatial filtering imposed by the gauge length^24^. The gauge length corresponds to the fiber segment over which strain is averaged, and this averaging suppresses short-wavelength components, attenuating modes with wavelengths shorter than twice the gauge length. Reliable guided wave measurements are not obtained in the Sandhill Road Bridge and the Yerba Buena Road Bridge. This is primarily due to structural and sensing constraints: the Sandhill Road Bridge is too short (29.3 m) to achieve reliable wavefield observations within the measurable frequency range, while the DAS gauge lengths for both bridges (16.3 m for Sandhill Road and 10 m for Yerba Buena Road) are too long, exceeding half of the dominant wavelength. SAFE simulations are also performed for the Byeongcheon 2 and Bakdal 2 bridges, while modeling for the McKee Road overpass is not conducted due to a lack of cross-sectional design drawings. Both the Byeongcheon 2 and Bakdal 2 bridges are composite structures with approximately uniform cross-sections, consisting of concrete slabs and steel beams (Supplementary Figs. 3c and 4c). Byeongcheon 2 uses steel I-beams, whereas Bakdal 2 features steel box girders. The SAFE simulated dispersion curves show good agreement with the observed data, particularly for the fundamental modes (Supplementary Figs. 3f and 4f). Although the cutoff frequencies of the higher-order modes differ, their overall shapes closely match the telecom-DAS-derived dispersion images. The corresponding guided-wave cross-sectional displacement patterns for the fundamental symmetric (*S*_0_) and antisymmetric (*A*_0_) modes are also obtained from the SAFE simulations (Supplementary Figs. 3g, h, and 4g, h). Despite the uniform cross-section, discrepancies between the measured and simulated dispersion curves emerge at frequencies above 100 Hz for both bridges. These mismatches likely arise from fine-scale structural features not captured in the SAFE models. For example, the presence of asphalt layers, which is difficult to characterize and omitted from the simulation, may contribute to the deviations observed in the high-frequency range.

### Estimation of quasi-static displacements

The reliable low-frequency sensitivity and dense spatial coverage of Telecom as Sensors measurements enable the estimation of bridge quasi-static displacements. In this study, quasi-static refers to low-frequency signals in the range of 0.1–1 Hz, primarily induced by vehicle loads. Traffic loads are the primary operational loads on bridges, and the resulting responses provide critical information for assessing performance, including load rating, fatigue-related degradation, and the potential for bridge weigh-in-motion applications^25–27^.

**Fig. 4:**
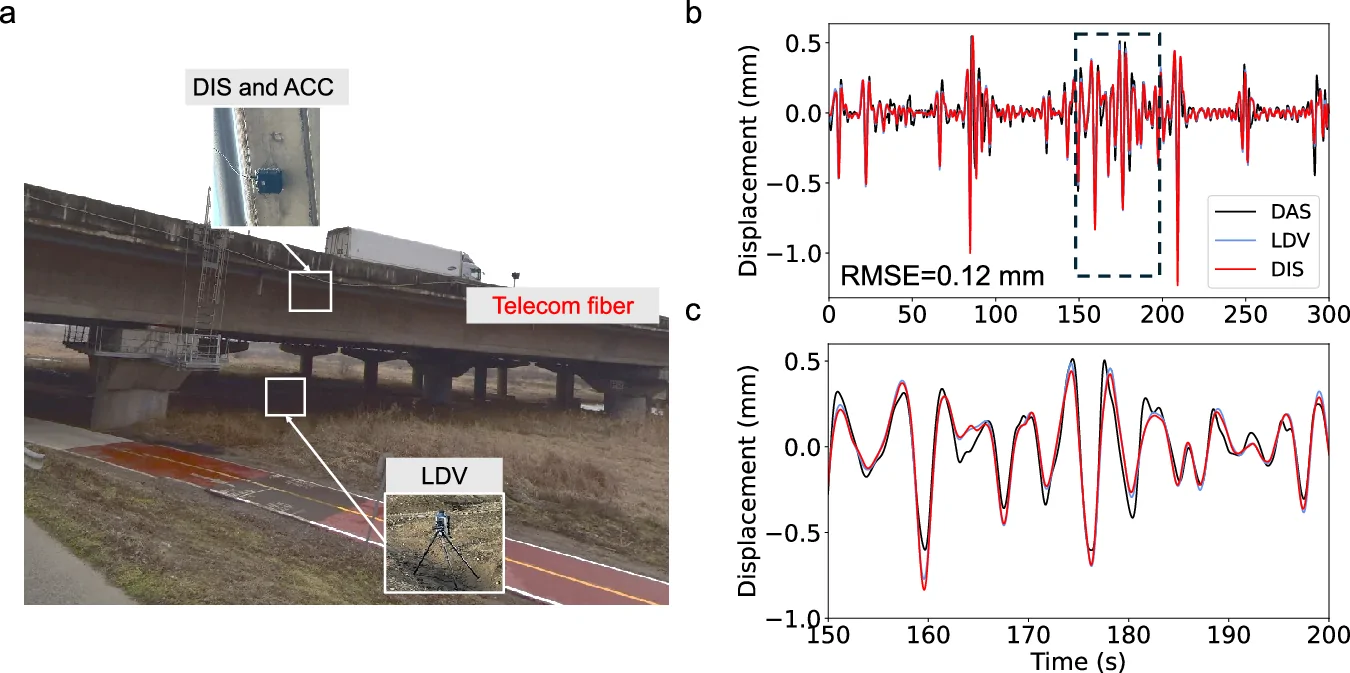
Quasi-static displacement estimation for the Miho River Bridge using Telecom as Sensors, validated against conventional sensors. **a** Field deployment showing the pre-existing telecom fiber-optic cable used for DAS, alongside an accelerometer (ACC), displacement sensor (DIS), and laser Doppler vibrometer (LDV) for ground-truth validation. Insets show close-ups of the LDV and DIS setups. A detailed view of the telecom fiber attachment is provided in Fig. 2b. **b** Comparison of vertical displacement time series over a 5-min window measured by DAS (black), LDV (red), and DIS (blue). DAS-based displacement estimation achieved a root mean squared error of 0.12 mm compared to LDV. **c** Zoomed-in view of the boxed region in (**b**), illustrating sub-millimeter agreement between DAS and validation sensing modalities in capturing quasi-static displacements.

To estimate displacement, we perform double spatial integration of the DAS strain data along the bridge span (see “Methods” for details). Our Telecom as Sensors-based displacement estimates at the mid-span are validated against conventional measurements from the displacement sensor and laser Doppler vibrometer (LDV; Fig. 4a and Supplementary Fig. 6a). For both the Miho River Bridge and the Bakdal 2 bridge, the Telecom as Sensors-based estimates achieve submillimeter accuracy, comparable to conventional dedicated sensors. Specifically, the root-mean-square error (RMSE) compared to the reference LDV is 0.12 mm for the Miho River Bridge (Fig. 4b) and 0.08 mm for the Bakdal 2 bridge (Supplementary Fig. 6b). In zoomed-in views of the displacement time series (Fig. 4c and Supplementary Fig. 6c), the deflection patterns clearly show downward displacements corresponding to vehicle-induced bending as loads traverse the span, which can also be used to infer vehicle weights. For the Byeongcheon 2 Bridge, quasi-static displacement estimates were not reliable due to poor coupling between the fiber and the bridge deck (Supplementary Fig. 7). For the bridges in the United States, validation sensors were not deployed due to logistical constraints associated with accessing the underside of the bridge structures.

### Analysis of dynamic responses

DAS using telecom fiber-optic cables coupled to bridges also enables reliable measurement of bridge dynamic responses. Liu et al.^28^ first performed modal analysis using Telecom as Sensors on a continuous three-span concrete bridge, successfully estimating resonant frequencies and mode shapes. Recently, Rodet et al.^29^ evaluated the feasibility of using dark fiber to monitor multiple bridges across an extended fiber network and captured daily variations in modal parameters using DAS on dark fiber. These modal parameters provide valuable insights into structural health: once environmental influences, such as thermal-induced stiffness changes, are accounted for, variations in modal characteristics signal structural changes or damage^30,31^. In this study, we extend the validation of Telecom as Sensors for dynamic response monitoring to additional bridges with diverse structural types (box girders and composite concrete-steel I-beams), span configurations, and lengths.

**Fig. 5:**
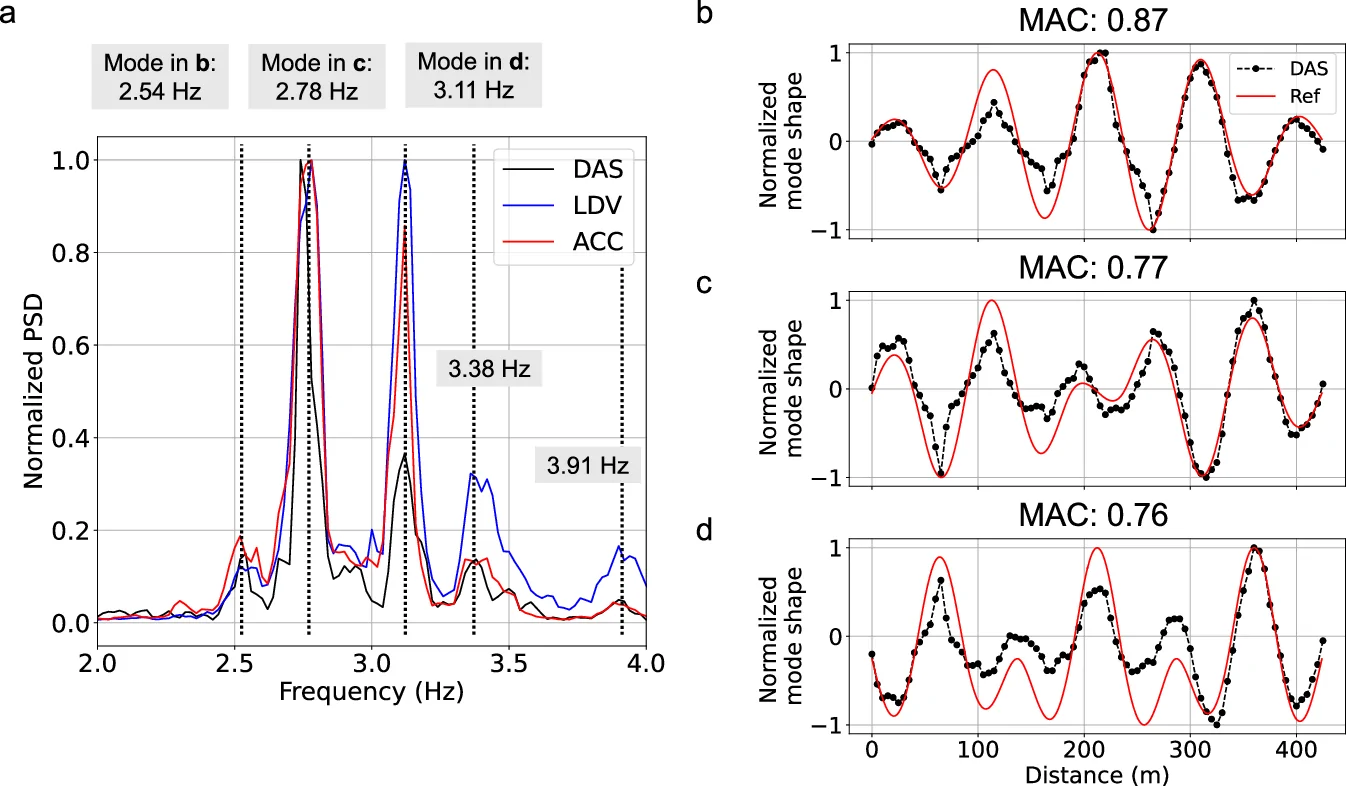
Frequency response and modal analysis for the Miho River Bridge captured using Telecom as Sensors, validated against conventional sensors and numerical modeling. **a** Normalized power spectral density (PSD) of dynamic responses measured by DAS (black), laser Doppler vibrometer (LDV, blue), and accelerometer (ACC, red), showing consistent identification of the first five modes at 2.54, 2.78, 3.11, 3.38, and 3.91 Hz. **b**–**d** Normalized mode shapes corresponding to the identified modes, derived from the DAS response using the frequency domain decomposition (FDD) method (see “Methods" for details) and compared with numerical results from OpenSees. The Modal Assurance Criterion (MAC) values for the first three modes are 0.87, 0.77, and 0.76, respectively. The high spatial resolution of DAS enables meter-scale reconstruction of mode shapes across the full length of the bridge span.

Reliable dynamic response measurements are obtained from all bridges except the Byeongcheon 2 bridge, where poor fiber coupling degrades signal quality (Supplementary Fig. 7). We validate the Telecom as Sensors-based measurements by comparing them with either numerical models^32^ based on available design drawings or experimental data from accelerometers and the LDV (see “Methods” for details). Power spectral density (PSD) is used to identify resonant frequencies, and FDD is applied to extract mode shapes from DAS signals (see “Methods” for details). Notably, DAS provides mode shapes with meter-scale spatial resolution, enabling fine-grained characterization of structural dynamics that is difficult to achieve with sparse conventional sensors. For the Miho River Bridge, we successfully identify the top five resonant frequencies with 100% accuracy with those obtained from the accelerometer and LDV (Fig. 5a). The mode shapes for the top three modes show strong agreement with numerical simulations, with Modal Assurance Criterion (MAC)^33^ values of 0.87, 0.77, and 0.76, respectively (Fig. 5b–d). The mode shapes of the two higher modes could not be reliably estimated because the gauge length in DAS acts as a spatial averaging window, which smooths out short-wavelength variations and reduces sensitivity to higher modes. For the Bakdal 2 bridge, PSD comparison also shows strong agreement between DAS and validation sensors, with the first two modes at 1.97 and 2.34 Hz consistently identified across all four structural partitions. The first mode shape achieves up to 0.93 MAC values (Supplementary Fig. 8). At the McKee Road Overpass and the Yerba Buena Road bridge, accelerometers deployed on the sidewalks provide field validation, with MAC values of 0.92 and 0.94 for their first mode, respectively (Supplementary Figs. 9 and 10).

Resonance is a fundamental phenomenon in structural dynamics. In plate-like structures, it occurs when left- and right-propagating waves traveling at finite speeds superpose to form standing waves^34^. At resonance, constructive interference amplifies vibration amplitudes, producing oscillations that remain localized rather than propagating through the structure. For a simplified bridge modeled as a Bernoulli–Euler (thin) beam, the fundamental-mode resonance occurs when the span length equals half the wavelength of propagating waves, $L=\frac{\lambda }{2}$, where *λ* = *v*/*f*, *L* is the span length, *v* the wave velocity, and *f* the frequency. This yields the resonance relationship *v* = 2*f*_0_*L*. Supplementary Fig. 11 shows measured dispersion spectra below 5 Hz for the Miho River, Bakdal 2, and McKee Road bridges. The Miho River Bridge has span lengths of 40 and 50 m, the Bakdal 2 bridge spans 50 m, and the McKee Road bridge spans 41 and 44 m. In each case, the identified resonance frequencies and corresponding standing-wave velocity ranges (2.54 Hz, 203–254 m/s; 1.97 Hz, 197 m/s; and 2.52 Hz, 207–222 m/s, respectively) align closely with the guided waves (*A*_0_ mode) observed at those frequencies. This agreement between modeled and measured signals confirms that standing waves arise from the superposition of left- and right-propagating guided waves.

**Fig. 6:**
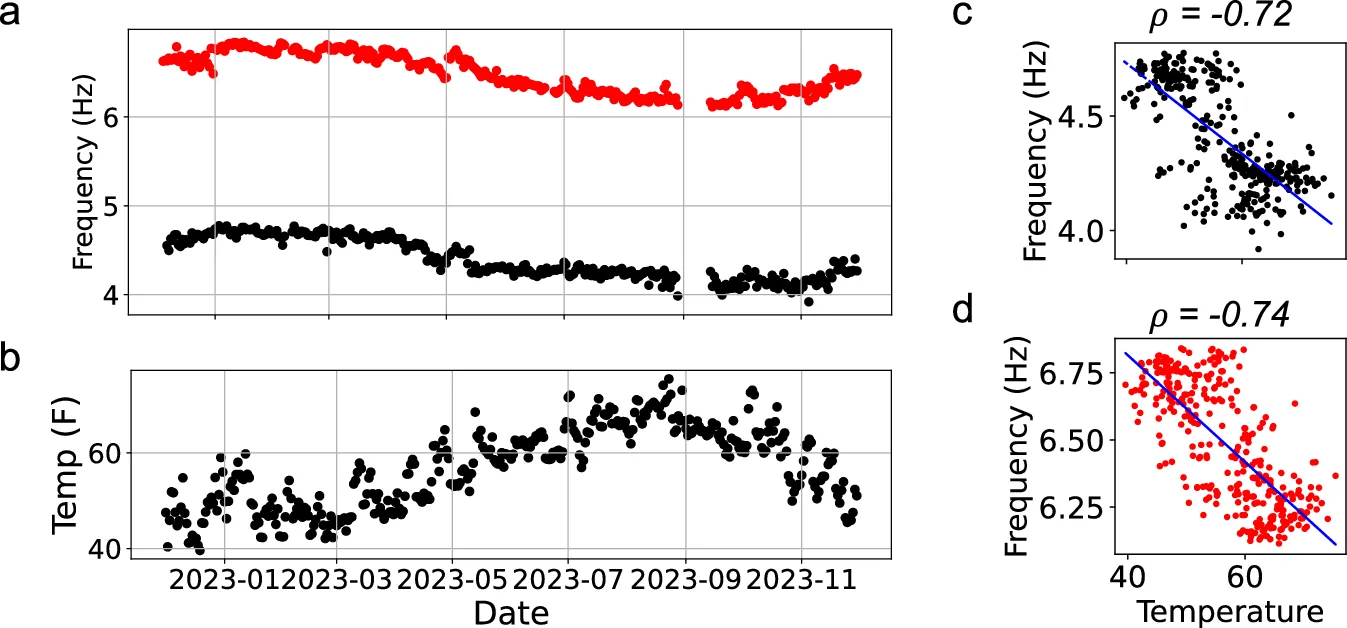
Long-term monitoring of resonant frequencies at the Sandhill Road Bridge and their correlation with temperature. **a** Time series of the first two resonant frequencies estimated using DAS on a pre-existing telecom fiber-optic cable over a year, exhibiting clear seasonal trends. **b** Daily ambient temperature recorded by the Stanford Weather Station during the same period. **c**,** d** Scatter plots illustrating the relationship between temperature and the first two resonant frequencies (338 samples), with linear regression fits and corresponding Pearson correlation coefficients (*ρ*). The results indicate a strong negative correlation, suggesting that resonant frequencies decrease with increasing temperature.

Telecom as Sensors also enables long-term and continuous monitoring of bridge dynamic response, as pre-existing fibers are naturally coupled with the structure. We have been continuously collecting DAS data from a telecom fiber coupled to the Sandhill Road Bridge in the City of Palo Alto for 1 year. The dynamic response of this bridge is validated by comparing PSDs derived from Telecom as Sensors and conventional accelerometers (Supplementary Fig. 12). Mode shape estimates were not obtained for this bridge because it is too short (29.3 m), while the gauge length of the DAS system is too long (16.3 m), limiting spatial resolution. The top two resonant frequencies were monitored from December 2022 to November 2023 (Fig. 6). These frequencies show strong negative correlations with ambient temperature, with Pearson correlation coefficients of −0.72 and −0.74, respectively. This enables quantification and correction for temperature-induced shifts in resonant frequencies, enhancing the reliability of long-term structural health assessment.

## Discussion

Using pre-existing telecom fiber-optic cables, we demonstrate the viability of DAS for comprehensive monitoring of bridge responses, including multi-mode guided wavefields, quasi-static displacements, and dynamic responses for modal analysis. Notably, many of the analyses presented here, including guided wavefield retrieval, resonant frequency identification, and mode shape extraction, are performed directly from the DAS data without requiring numerical models or reference sensors. In this study, the numerical models and conventional sensors were used exclusively for validation. Absolute displacement estimation requires a one-time calibration based on either a reference measurement or design drawings, provided that the neutral axis location is known. In practice, combining Telecom as Sensors with a limited number of conventional instruments could further improve validation and interpretation, particularly for capturing response components that are not aligned with the fiber axis.

A key advantage of this approach lies in its scalability and cost-effectiveness. Traditional monitoring systems require the installation and long-term maintenance of dense sensor networks, which can be prohibitively expensive for large-scale deployment. For example, the monitoring system on the Bill Emerson Memorial Bridge in Missouri cost approximately $1.3 million for 86 accelerometer channels (approximately $15,000 per channel)^35^. In contrast, Telecom as Sensors leverages existing telecom fibers that eliminate the installation expenses of on-site sensing systems. While a DAS interrogator requires a significant upfront investment, this cost can be amortized over tens of kilometers of fiber and across multiple bridges monitored simultaneously, resulting in a per-channel cost of only a few dollars per meter of fiber, which is substantially lower than that of dedicated sensor networks^15^. Also, unlike traditional sensor networks, telecom fibers require no additional long-term maintenance since they are already maintained to serve communication functions. Access to fiber is therefore critical. In practice, agencies may also lease existing dark fibers at relatively low cost, providing a practical and economically attractive model for scaling the technology across transportation networks. Notably, a single fiber can span multiple bridges. For instance, the 14-km telecom fiber we used at Cheongju Expressway passes through seven bridges. All DAS channels along a fiber are connected and inherently synchronized, eliminating the need for costly cabling or wireless connectivity. As a result, Telecom as Sensors provides potential cost savings of orders of magnitude, enabling scalable and economically viable monitoring across large bridge networks. Beyond cost efficiency, the high spatiotemporal resolution of DAS provides unique measurement capabilities. It enables meter-scale resolution of bridge mode shapes, demonstrating that telecom fiber networks can be used for modal analysis, and it further enables continuous monitoring of guided wavefields along the bridge superstructure and quasi-static displacement estimation from the same sensing system. This combination of capabilities, together with validation across six bridges and year-long monitoring, distinguishes the present work from earlier telecom-fiber studies.

Minor discrepancies remain between our results and those obtained from validation sensors or numerical simulations, and several factors, including cable-to-bridge coupling strength, DAS interrogator settings, and environmental noise, influence the reliability of DAS-based measurements. The bridge examples in this study further highlight these practical limitations: at Sandhill Road, the short span relative to the DAS gauge length prevented reliable guided wavefield and mode shape retrieval, while at Yerba Buena Road, the 10 m gauge length similarly constrained guided wave observations. At Byeongcheon 2, poor fiber-bridge coupling degraded the signal quality, precluding reliable quasi-static displacement recovery and weakening modal-analysis performance. Future research should systematically characterize the impact of these factors, particularly coupling conditions and sensing configuration. Ad hoc signal processing and calibration methods could be developed to mitigate their effects^36,37^. Additionally, DAS generates large volumes of high-rate data, necessitating investment in efficient signal processing pipelines, storage systems, and visualization methods.

Yet, despite these addressable limitations, our method has strong scaling potential with minimal cost. All measurements in this study were obtained using pre-existing telecom fibers without any additional sensor installation or maintenance. Telecom as Sensors thus provides a cost-effective, scalable, and maintenance-free platform for continuous structural monitoring on a large scale. Because telecom fibers are already embedded in diverse infrastructure systems, including roads, tunnels, pipelines, and power grids, the same approach could be extended to monitor these structures as well. In the longer term, time-lapse monitoring could extend beyond resonant frequency tracking to capture changes in wave propagation properties through successive daily measurements, thereby revealing localized damage. Because stable guided wavefields can be retrieved within 20 hours of ambient traffic recording (Supplementary Fig. 13), the approach is compatible with routine daily monitoring. Localized structural degradation, such as cracking or section loss, would produce spatially concentrated anomalies in the dispersion characteristics, in contrast to temperature-induced changes, which tend to be spatially uniform; these localized anomalies could then be imaged using guided-wave full-waveform inversion^38^.

## Methods

### Description of monitored bridges

This study involves six roadway bridges located in South Korea and the United States. The monitored structures span a wide range of lengths from 29.3 to 700 m, structural types, materials, and span configurations, enabling broad validation of the proposed sensing system under realistic operating conditions. Data collection durations ranged from several days to a year, depending on site access. Figure 2 summarizes the structural characteristics, span arrangements, and sensing configurations for all monitored bridges. More detailed design drawings for the monitored bridges are not included because these bridges are operational infrastructure, and the corresponding documents are subject to sharing restrictions.

In South Korea, three highway bridges were monitored. The Miho River Bridge, located in Cheongju, Chungcheongbuk-do, is a 430 m long concrete box girder structure configured as a continuous 9-span system. The Byeongcheon 2 bridge, in Cheonan, Chungcheongnam-do, is a 240 m long composite bridge comprising steel I-beams with a concrete deck and a continuous 11-span configuration. The Bakdal 2 bridge, located in Anyang, Gyeonggi-do, is the longest among the three, with a total length of 700 m. It comprises composite steel box girders with a concrete deck and has a partitioned span arrangement of four segments: 3-span, 4-span, 4-span, and 3-span.

In California, three additional bridges were studied. The McKee Road Overpass in San Jose, California, is an 84.3-m-long concrete box girder structure with a continuous 2-span configuration. The Yerba Buena Road Bridge, also in San Jose, California, spans 96.9 m with a continuous 3-span concrete box girder design. The Sandhill Road Bridge in Palo Alto, California, is a single-span, 29.3 m long bridge with a skew angle of 30°, constructed using steel I-girders and a concrete deck. This site was monitored through multiple campaigns for nearly one year, enabling repeated and long-term measurements.

### DAS data acquisition

DAS measurements were conducted on all six bridges using pre-existing telecom optical fibers coupled to the bridge superstructures. For bridges in South Korea, interrogators were installed at Korea Expressway Corporation offices in Cheongju and Siheung. The DAS data were collected by an AP Sensing N51 interrogator from telecom fiber-optic cables installed in 1997 for communication between expressway offices. These cables were housed in air-filled PVC conduits and secured to the bridge deck with metal strap clamps. The conduits were clamped to the side of the box girder for the Miho River Bridge (Fig. 3b), to the side of the concrete slab for the Byeongcheon 2 bridge (Supplementary Fig. 3b), and to the top surface of the concrete slab for the Bakdal 2 bridge (Supplementary Fig. 4b). At the Sandhill Road Bridge, DAS data were collected by an Optasense ODH3 interrogator from the Stanford campus fiber array^39^, which consists of a loosely deployed optical cable housed in an air-filled PVC conduit. The cable extends approximately 45 km, beginning in central Stanford campus and running beneath portions of Palo Alto, Menlo Park, and Redwood City. Within the bridge span, the conduit is mechanically clamped to a steel I-girder. At the Yerba Buena Road Bridge and the McKee Road overpass, DAS data were collected by an Optasense Quantx interrogator from a 50-km-long telecom fiber operated by the City of San Jose^16^, with the interrogator installed in the city hall server room. At the Yerba Buena Road Bridge, the cable was routed along the top surface of the bottom flange of the concrete box girder. The location and coupling method of the telecom cable at the McKee Road overpass were not accessible and could not be visually confirmed.

The quality and interpretability of DAS measurements depend on the mechanical coupling between the telecom cable and the bridge, which governs strain transfer from the structure to the fiber. Because the fiber is coupled through a conduit that is mechanically clamped to the structure, rather than being directly bonded to the bridge, spatial variability in clamp tightness and conduit contact can produce along-cable variations in overall measurement quality. The Byeongcheon 2 bridge illustrates that inadequate coupling (Supplementary Fig. 7) can preclude reliable recovery of quasi-static displacement and degrade modal analysis performance. In contrast, the successful results from the other five bridges indicate that clamp-based coupling, when reasonably tight and continuous, is sufficient for the dynamic response, guided-wavefield, and quasi-static displacement analyses presented here.

The DAS configurations vary across sites in gauge length, channel spacing, and sampling frequency, depending on storage and equipment availability (Fig. 2). Gauge length and channel spacing involve inherent trade-offs: longer gauge lengths generally provide higher signal-to-noise ratios but lower spatial resolution, whereas finer channel spacing improves spatial resolution at the expense of greater data redundancy and storage demand. In South Korea, DAS signals of the Miho River Bridge, Byeongcheon 2 bridge, and Bakdal 2 bridge were recorded in strain rate, with a gauge length of 4.9 m and channel spacing of 1.2 m. The Miho River and Byeongcheon 2 bridges were sampled at 1000 Hz, while the Bakdal 2 bridge was sampled at 500 Hz. In the United States, strain measurements were acquired. The McKee Road Overpass used a 2-m gauge length and 1-m channel spacing at 200 Hz. The Yerba Buena Road Bridge used a 10-m gauge length and 1-m channel spacing at 200 Hz. The Sandhill Road Bridge used a 16.3-m gauge length and 8.2-m channel spacing at 250 Hz. The sampling frequencies used in this study are sufficient to capture modal parameters and quasi-static displacement components and therefore exceed the Nyquist requirement by a wide margin. For guided wave analysis, the sampling frequency defines the upper bound of the observable frequency content. In practice, however, the effective upper frequency limit in DAS is also constrained by the gauge length, which acts as a spatial filter^24^.

### Description of validation sensing systems

The quasi-static displacements and dynamic responses derived from DAS measurements are validated using multiple conventional sensing systems across the test bridges (Fig. 2). For the three bridges in South Korea, quasi-static displacement measurements are validated using a custom displacement sensor and a Polytec RSV-150 laser Doppler vibrometer (LDV). The displacement sensor was installed beneath the mid-span of the target span and integrates three components: a micro-electro-mechanical systems (MEMS) accelerometer (ADXL355, Analog Devices Inc.), a 60 GHz frequency-modulated continuous-wave radar (BGT60TR13C, Infineon), and an embedded micro-processing unit. The integrated MEMS accelerometer also served to validate dynamic responses and natural frequency estimates of the bridges. Key specifications of the MEMS accelerometer include a noise density of 22.5 μg$/\sqrt{\,{\mbox{Hz}}\,}$, repeatability of ±3 mg, non-linearity below 0.1%, and selectable full-scale ranges of ±2 g, ±4 g, and ±8 g (set to ±2 g in this study). After an automatic calibration procedure, which is used to identify the most stable natural reflector and determine the line-of-sight-to-displacement conversion factor, the sensor entered continuous monitoring mode. It combines radar phase data with accelerometer readings to estimate displacement at a sampling rate of 100 Hz^40,41^. Additionally, the LDV supports 16 sensitivity ranges from 1 μ m/V to 100 mm/V; a 1 mm/V setting was used in this study. The instrument provides an analog output of ±10 V via BNC connectors, with a resolution of 0.3 nm and a measurement bandwidth of 0 Hz to 25 kHz. The LDV operates at a wavelength of 1550 nm and was configured to sample at 100 Hz.

For the bridges in California, dynamic responses are validated using PCB 354C03 piezoelectric accelerometers mounted on the bridge deck. These sensors have a sensitivity of 100 mV/g, a measurement range of ±50 g (peak), and a spectral noise density of 100 μg$/\sqrt{\,{\mbox{Hz}}\,}$ at 1 Hz. On the McKee Road overpass and the Yerba Buena Road Bridge, we conducted roving sensor tests. This involves moving accelerometers across predefined positions on the bridge to collect spatially distributed vibration data for mode shape identification. The sensor locations for the roving tests are shown as red triangles in Supplementary Figs. 9b and 10b.

### Retrieving guided wavefields from DAS data

To extract coherent guided wavefields from Telecom as Sensors recordings, we use ambient noise interferometry^21,42^ to construct Virtual Source Gathers (VSGs) from traffic-induced ambient noise signals. This passive imaging approach enables the retrieval of empirical Green’s functions between distributed sensing points without requiring active sources, making it particularly suitable for operational bridges where controlled excitation is impractical due to safety and logistical constraints.

We process the continuous DAS strain-rate data recorded along the bridge using the following workflow. In the pre-processing steps, the raw signals are detrended and high-pass filtered at 1 Hz to suppress quasi-static components dominated by vehicle-induced deformation. The filtered waveform is segmented into 10-s windows, and each segment undergoes time-domain normalization and spectral whitening to mitigate amplitude bias and equalize spectral content across frequencies. For each pair of DAS channels (*x*_*a*_, *x*_*b*_), the empirical Green’s function is approximated by cross-correlating their frequency-domain representations:

1 $${C}_{ab}(\tau )={{{{\mathcal{F}}}}}_{a}^{*}(\omega ){{{{\mathcal{F}}}}}_{b}(\omega ),$$

where *C*_*ab*_(*τ*) denotes the empirical Green’s function between channels *x*_*a*_ and *x*_*b*_, and ${{{\mathcal{F}}}}$ is the Fourier transform of the DAS recordings. The cross-correlations obtained from all time windows are then linearly stacked to enhance coherent energy from stationary signals (such as guided waves) while suppressing incoherent noise from random or off-path sources. By designating one DAS channel as a virtual source and treating the remaining channels as receivers, we generate VSGs that capture the propagation of guided waves in the bridge. Figure 3d, e, and Supplementary Figs. 3d, e, 4d, e, 5c show the retrieved wavefields from the Miho River Bridge, Byeongcheon 2 bridge, Bakdal 2 bridge, and McKee Road overpass, respectively. We obtain dispersion images from the VSGs using the phase-shift method^43^, as shown in Fig. 3f and Supplementary Figs. 3f, 4f, 5d.

To illustrate the effectiveness of stacking in enhancing coherent signals, we track the evolution of a DAS trace located 300 m from the virtual source on the Miho River Bridge. Supplementary Fig. 13 shows the trace at different stacking durations and the corresponding improvement in signal-to-noise ratio (SNR). SNR is computed by comparing the energy within the 1–2 s signal window against that in a subsequent noise window. The results indicate a progressive improvement in SNR over time, with the guided wave signal reaching convergence after approximately 20 hours of stacking.

### Numerical modeling of guided wave modes

We perform guided wave mode analysis using the semi-analytical finite element (SAFE) method^23^. The SAFE approach applies finite element discretization to the bridge cross-section and introduces an analytical wave propagation term, *e*^*i*(*ωt*−*kz*)^, along the longitudinal (*z*) axis, where *ω* is the angular frequency, *t* is time, and *k* is the wavenumber. Compared to full three-dimensional finite element modeling, SAFE reduces the problem to a two-dimensional domain, significantly improving computational efficiency while accurately capturing guided wave behavior in structures with constant geometry along one axis. This method assumes (1) an infinitely long structure, (2) structural uniformity along the *z*-direction, and (3) harmonic wave propagation, such that the displacement field can be written as ${{{\bf{u}}}}(x,y,z,t)=\hat{{{{\bf{u}}}}}(x,y){e}^{i(kz-\omega t)}$, where $\hat{{{{\bf{u}}}}}(x,y)$ is the cross-sectional mode shape. Here, mode shape $\hat{{{{\bf{u}}}}}(x,y)$ denotes the guided-wave displacement distribution over the bridge cross-section obtained from the SAFE simulation, rather than a global structural resonance mode shape of the entire bridge. Based on the finite element discretization of the cross-section, the element stiffness and mass matrices are assembled to formulate the following eigenvalue problem:

2 $$\left({k}^{2}[{{{{\bf{K}}}}}_{2}]+k[{{{{\bf{K}}}}}_{1}]+[{{{{\bf{K}}}}}_{0}]-{\omega }^{2}[{{{\bf{M}}}}]\right){{{\bf{U}}}}=0,$$

where **U** are the vectors of displacement amplitudes, [**K**_0_], [**K**_1_], and [**K**_2_] are stiffness matrices, and [**M**] is the mass matrix. Solving this eigenvalue problem yields the dispersion relationship between *ω* and *k*. The eigenvector in **U** associated with each eigenvalue represents the corresponding mode shape.

In our implementation, the bridge cross-section is modeled in COMSOL Multiphysics using the Solid Mechanics module under a two-dimensional plane strain assumption. Cross-sectional geometries are extracted from available design drawings, and material properties, including shear wave velocity (*c*_*s*_), compressional wave velocity (*c*_*p*_), and density (*ρ*), are assigned to each structural component. Nominal material properties are used for both steel and concrete^44–46^. For steel, we use *c*_*s*_ = 3200 m/s, *c*_*p*_ = 5900 m/s, and *ρ* = 7850 kg/m^3^. For concrete, we assign *c*_*s*_ = 2350 m/s, *c*_*p*_ = 4200 m/s, and *ρ* = 2400 kg/m^3^, which are applied to all bridges except Bakdal 2. For the Bakdal 2 bridge, we assign a lower shear wave velocity of *c*_*s*_ = 2000 m/s, which provides the best match with the observed dispersion spectrum. This adjustment likely reflects differences in concrete quality or aging effects. Dispersion curves are computed by sweeping across 250 frequency steps from 2 to 500 Hz. For each frequency step, the eigenvalue problem is solved to obtain the corresponding wavenumber *k*, from which the phase velocity is derived. The dispersion curves computed from SAFE modeling are overlaid with the observed data in Fig. 3f and Supplementary Figs. 3f,4f for the Miho River Bridge (mid-span cross-section), Byeongcheon 2 bridge, and Bakdal 2 bridge, respectively. The corresponding guided-wave cross-sectional displacement patterns for the fundamental antisymmetric (*A*_0_) and symmetric (*S*_0_) modes are shown in Fig. 3g, h, and Supplementary Figs. 3g, h, 4g, h. SAFE modeling was not conducted for the remaining bridges due to the lack of cross-sectional design drawings.

### Estimating bridge quasi-static displacement

We estimate bridge quasi-static displacement (defined in this study as structural responses in the 0.1–1 Hz frequency range) using distributed measurements of axial strain along the bridge girder obtained from Telecom as Sensors. Under vehicle loading, the response is primarily governed by flexural deformation, enabling vertical displacement to be inferred from the strain profile using Euler–Bernoulli beam theory. For a beam undergoing bending, the axial strain, *ϵ*(*x*), at a vertical offset *z* from the neutral axis is related to the displacement *w*(*x*) by:

3 $$\epsilon (x)=-z\cdot \kappa (x)=-z\cdot \frac{{d}^{2}w(x)}{d{x}^{2}},$$

where *x* is the longitudinal coordinate along the bridge, *κ*(*x*) is the curvature, and *z* is the distance from the neutral axis to the DAS fiber, assumed constant across the sensing region. The displacement *w*(*x*) can be recovered via double spatial integration of the strain:

4 $$w(x)=-\frac{1}{z}\int\int\epsilon (x)d{x}^{2}+{C}_{1}x+{C}_{2}$$

where *C*_1_ and *C*_2_ are integration constants determined by boundary conditions. Since *z* cannot be directly measured on site, we compute a scaling factor by dividing the displacement sensor reading by the DAS-estimated displacement at the beginning of the experiment. This calibration is required only once and therefore does not affect scalability. For the South Korean bridges, where the DAS system measures strain rate, we first integrate the strain-rate signal in time to obtain axial strain. We then apply numerical double integration using the trapezoidal rule^47^ in spatial across the bridge span. To resolve the integration constants, we impose zero displacement and zero slope at the bridge supports. The resulting displacement signals are bandpass filtered between 0.1 and 1 Hz to isolate quasi-static components. The estimated vertical displacements at mid-span locations are validated against ground-truth measurements obtained from the validation displacement sensor and LDV (Fig. 4 and Supplementary Fig. 6).

### Modal analysis using frequency domain decomposition

We extract the bridge’s mode shapes using the FDD method^48,49^. This output-only technique is well-suited for analyzing ambient vibrations, as it operates directly on the measured response signals and does not require knowledge of the input excitation. Its standard formulation assumes broadband, approximately stationary, and spatially uncorrelated excitation. Although these assumptions are only approximately satisfied under traffic loading, vehicle-induced excitation is typically sufficiently broadband for practical application. The FDD method begins by computing the power spectral density (PSD) matrix ${\hat{{{{\bf{G}}}}}}_{yy}(j\omega )={\mathbb{E}}[Y(j\omega )Y{(j\omega )}^{H}]$, where $Y(j\omega )={[{Y}_{1}(j\omega ),\cdots,{Y}_{M}(j\omega )]}^{T}$ are Fourier transforms of the *M*-channel DAS signals, *j* is the imaginary unit, and *ω* is the angular frequency. At each discrete frequency *ω* = *ω*_*i*_, a singular value decomposition (SVD) of the PSD matrix is performed:

5 $${\hat{{{{\bf{G}}}}}}_{yy}(j{\omega }_{i})={{{{\bf{U}}}}}_{i}{{{{\bf{S}}}}}_{i}{{{{\bf{U}}}}}_{i}^{H},$$

where **S**_*i*_ is a diagonal matrix containing the singular values *s*_*ik*_, **U**_*i*_ is a unitary matrix containing the corresponding singular vectors *u*_*ik*_, and the superscript *H* denotes the conjugate transpose. Near a resonance frequency associated with the *i*-th mode (or a closely spaced mode), one singular value dominates the spectrum. In such cases, the first singular vector, *u*_*i*1_, provides an estimate of the corresponding mode shape. The modal analysis results for the Miho River Bridge and the Bakdal 2 bridge are shown in Fig. 5 and Supplementary Fig. 8, respectively.

For the DAS signals, all available output channels are used to compute the PSD matrix and estimate mode shapes with meter-scale spatial resolution. This dense spatial sampling provides a high-resolution view of the mode shapes along the bridge superstructure. Note that because gauge length varies across sites, the achievable spatial resolution of mode shapes differs between bridges: shorter gauge lengths permit the identification of higher-order modes with shorter spatial wavelengths. We also deployed accelerometers on the McKee Road overpass and the Yerba Buena Road Bridge as validation sensors. These accelerometers were installed on the bridge sidewalks and used to provide independent reference measurements for modal analysis. To increase spatial coverage with the limited number of validation sensors, we conducted roving tests^50^ by sequentially repositioning the accelerometers along the bridge span, ensuring overlap between configurations for stitching mode shapes. The roving test results for the McKee Road overpass and the Yerba Buena Road Bridge are shown in Supplementary Figs. 9b and 10b, respectively.

For the FDD and PSD analyses, 5 min of data were used for both DAS and validation sensors, with FFT-based spectral estimates computed from five segments using 50% overlap. For the Miho River and Bakdal 2 bridges, the DAS data were low-pass filtered and downsampled to 20 Hz, and the LDV and accelerometer data, originally sampled at 100 Hz, were downsampled to 20 Hz. For the McKee Road and Yerba Buena Road bridges, the DAS data were low-pass filtered and downsampled from 200 to 50 Hz, while the accelerometer data, originally sampled at 2048 Hz, were downsampled to 64 Hz. The 5 min window was used throughout to maintain a uniform analysis framework across all bridges and sensing modalities and to remain compatible with the shorter 5–10 min LDV and accelerometer acquisition windows, thereby ensuring direct comparability between DAS and reference-sensor results across all case studies.

### Numerical modeling of bridge mode shapes

For the Miho River Bridge and the Bakdal 2 bridge, where reliable mode shapes were obtained from DAS measurements but validation sensors were unavailable due to the high cost and complexity of achieving sufficient spatial resolution, we validated the experimental results using numerical models developed in OpenSees^32^. In both cases, the main girders were modeled as linear elastic Euler–Bernoulli beams with a spatial discretization of 1 m. Span configurations and cross-sectional properties were derived from as-built structural drawings. Material properties for concrete included a Young’s modulus of 35 GPa and a density of 2400 kg/m^3^. For steel elements, we used a Young’s modulus of 205 GPa, a shear modulus of 79 GPa, and a density of 7850 kg/m^3^. Lumped masses were assigned to each beam node based on tributary length and sectional mass per unit length. For the Bakdal 2 bridge, which comprises multiple isolated partitions separated by joints, each partition was modeled independently to reflect its structural boundaries. To model the connection between the girder centerline and bearing locations, we introduced rigid arms at each support station, linking the neutral axis of the girder to the top of the bearing stack. Each bearing was then represented by a pair of zero-length spring elements connecting the top and bottom nodes of the bearing, where the bottom nodes were fully fixed to simulate the condensed substructure. Modal analysis was conducted through eigenvalue extraction to obtain mode shapes. This modeling framework enables direct comparison between simulated and measured mode shapes through the computation of the Modal Assurance Criterion^33^ (MAC; Fig. 5b–d and Supplementary Fig. 8b).

## Supplementary information

Supplementary Information

Transparent Peer Review File

## Source data

Source Data

## Data Availability

All the data generated in this study have been deposited in the GitHub repository^51^. Supplementary Information is available for this paper. Source data are provided with this paper.

## Code availability

The code for ambient noise interferometry is adopted from ref. ^52^ and available at^53^. The implementation of frequency domain decomposition is adopted from ref. ^54^. All figure generation scripts are available in the GitHub repository^51^.
