# Supplementary material for: Bridge health monitoring using existing telecommunication fiber-optic networks
Source: Nat Commun. 2026 Aug 21;17:9966. doi: 10.1038/s41467-026-76935-0 (PMC13586185; doi:10.1038/s41467-026-76935-0)
Supplement: Supplementary file 1 [file 41467_2026_76935_MOESM1_ESM.pdf]

Supplementary Information: Bridge Health Monitoring Using Existing Telecom Fiber-Optic Networks

Jingxiao Liu[*1,2], Jatin Aggarwal[3,a], Doyun Hwang[3,a], Jigu Lee[4], Yuyan Wu[3], Fu Yin[5], Haipeng Li[6], Paolo Santi[1,7,b], Hoon Sohn[4,b], Biondo Biondi[6,b], Carlo Ratti[1,8,b], and Hae Young Noh[3,b]

[1]Senseable City Laboratory, Massachusetts Institute of Technology, Cambridge, MA 02139, USA

[2]School of Civil and Environmental Engineering, Nanyang Technological University, Singapore, 639798

[3]Department of Civil and Environmental Engineering, Stanford University, Stanford, CA 94305, USA

[4]Department of Civil and Environmental Engineering, Korea Advanced Institute of Science and Technology, Daejeon 34141, Republic of Korea

[5]Department of Earth, Environmental, and Planetary Sciences, Rice University, Houston, TX 77005, USA

[6]Department of Geophysics, Stanford University, Stanford, CA 94305, USA

[7]Istituto di Informatica e Telematica del CNR, Pisa, Italy

[8]Dipartimento ABC, Politecnico di Milano, Italy

[a]These authors have equal contribution.

[b]These authors jointly supervised this work.

August 5, 2026

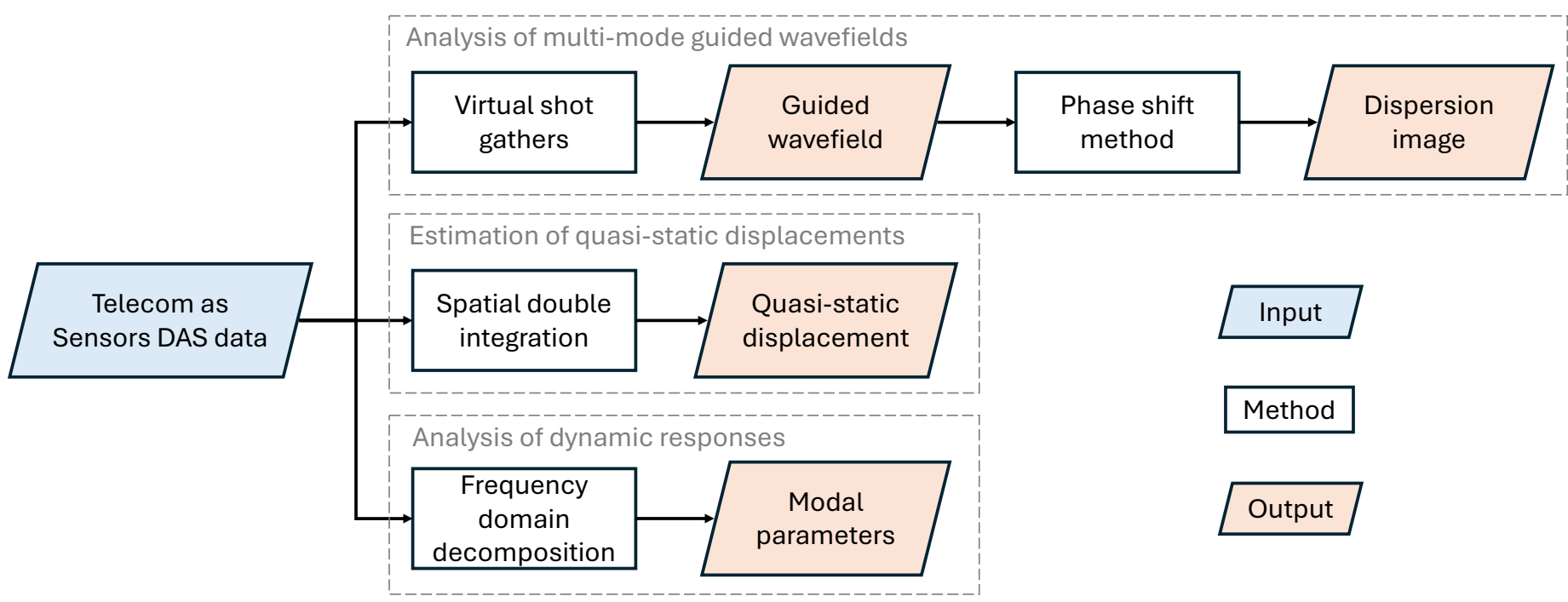

Supplementary Fig. 1: **Schematic overview of the analysis framework applied to Telecom as Sensors DAS data for bridge monitoring.** Telecom as Sensors DAS data are analysed through three pipelines: guided-wave analysis, quasi-static displacement estimation, and dynamic-response analysis. Guided-wave analysis uses ambient-noise interferometry to construct virtual source gathers (VSGs), from which guided wavefields and dispersion images are derived. Quasi-static displacement is estimated by spatial integration of the DAS strain data. Dynamic responses are analyzed using power spectral density and frequency domain decomposition to identify resonant frequencies and extract mode shapes.

*jingxiao@mit.edu

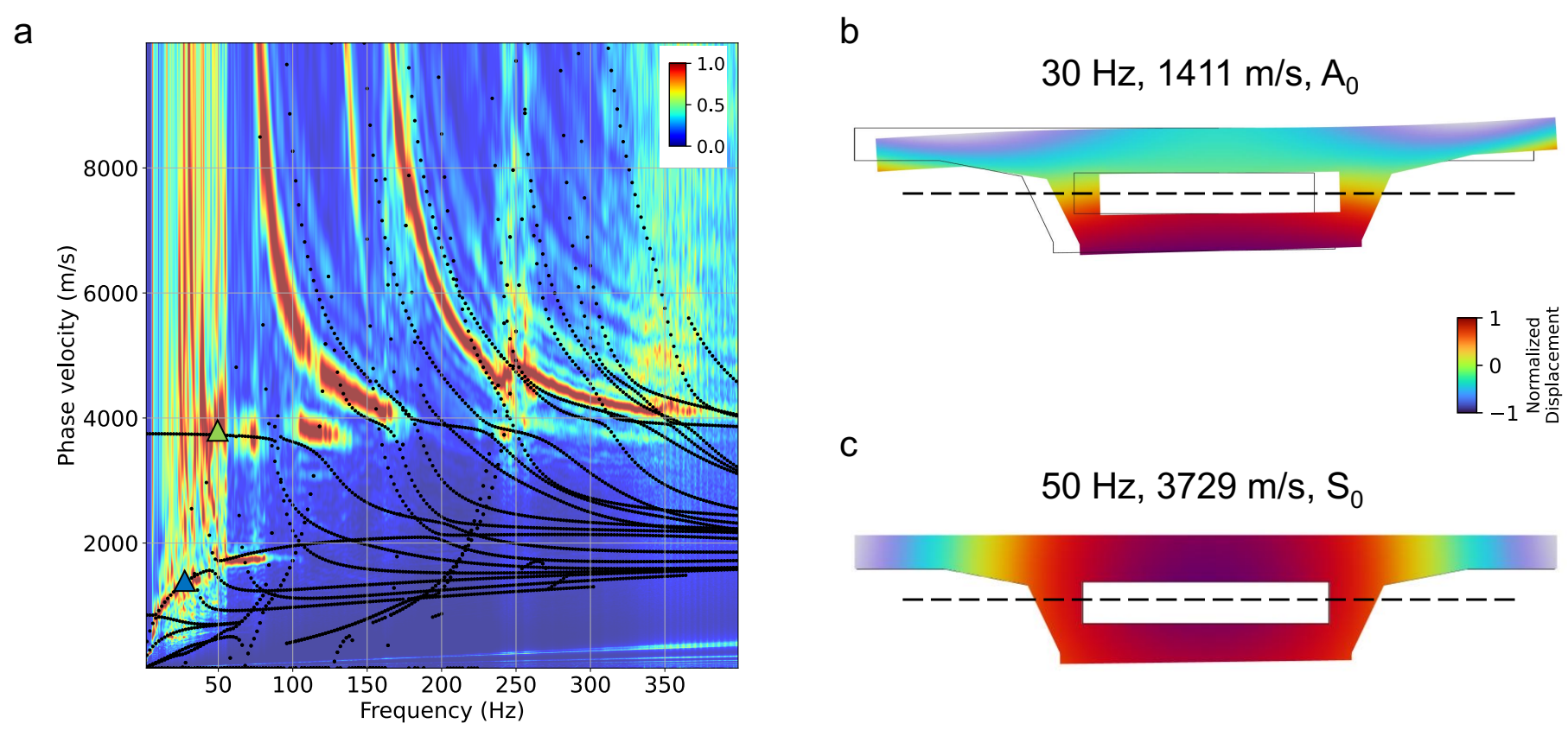

Supplementary Fig. 2: **Dispersion image and mode shapes for the terminal segment cross section of the Miho River bridge. a** Dispersion image derived from the wavefield in Fig. 2d. The image is normalized by frequency to enhance visibility across the spectrum and is overlaid with dispersion curves obtained from the SAFE-based guided wave modeling (black dots; see "Methods" for details). The mode shapes of A_0 and S_0 at 30 Hz and 50 Hz, respectively, are shown in panels **b** and **c**, with blue and green triangles indicating each mode in panel **a**. **b** Out-of-plane displacement mode shape for the A_0 mode at 30 Hz, with a propagation phase velocity of 1411 m/s. The displacement field is antisymmetric with respect to the central dashed line. **c** Out-of-plane displacement mode shape for the S_0 mode at 50 Hz, with a propagation phase velocity of 3729 m/s. The displacement field is symmetric with respect to the central dashed line.

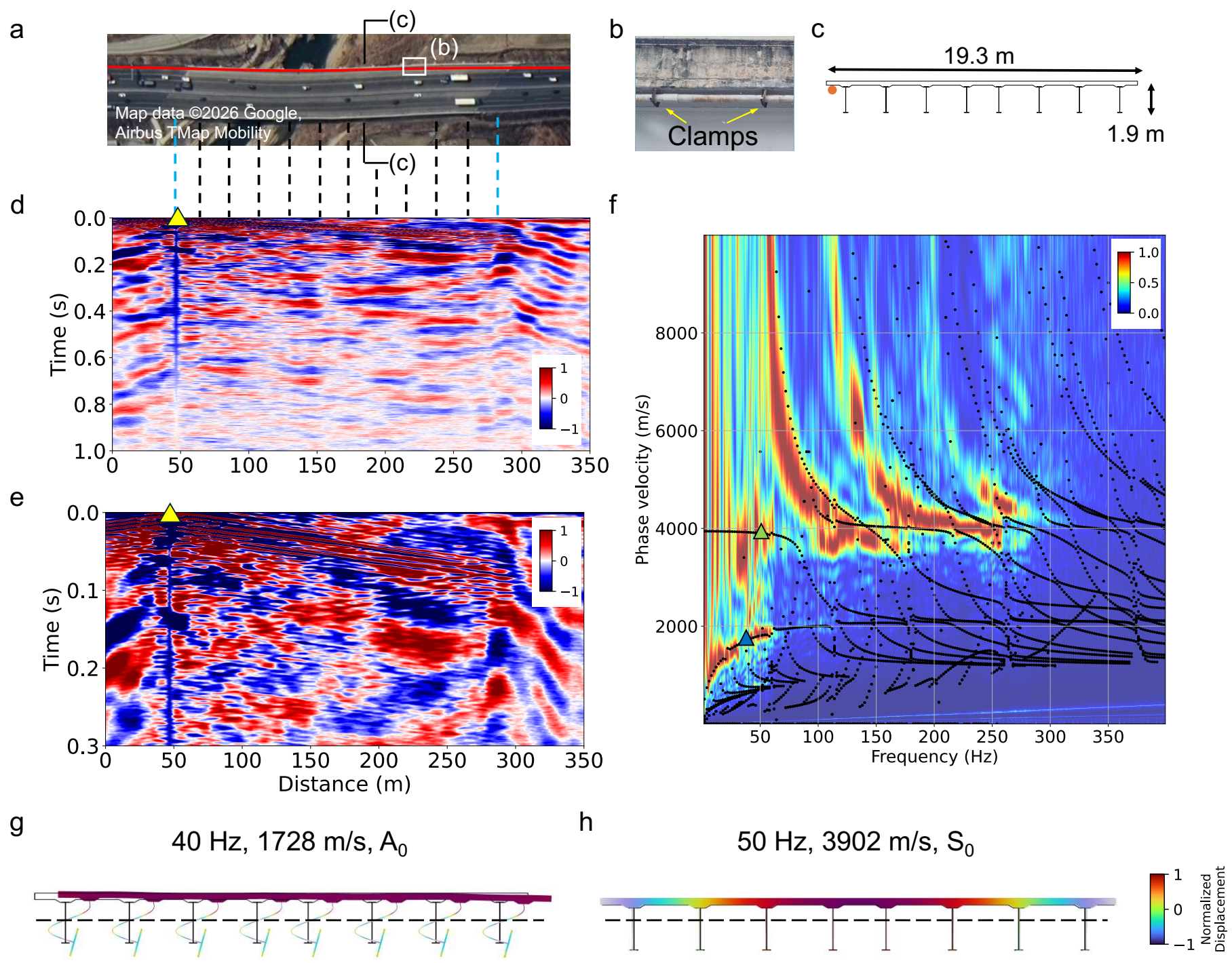

Supplementary Fig. 3: **Analysis of guided wavefields in the Byeongcheon 2 bridge using Telecom as Sensors.** **a** Aerial image of the bridge from Google Earth, with the red line indicating the route of the pre-existing telecom fiber-optic cable. Vertical dashed lines denote the locations of bridge piers (black) and expansion joints (blue). **b** Photo showing the side of the bridge where the telecom cable is mechanically coupled to the structure using clamps spaced every 2.4 meters. **c** Cross-section of the bridge. The orange dot indicates the location of the mounted fiber. **d** Virtual source gather showing the propagation of guided waves along the bridge. The data are normalized for visualization. The yellow triangle marks the virtual source location. **e** Zoomed-in view of (**d**) from 0 to 0.3 seconds, showing dispersive higher-frequency guided waves with faster propagation speeds. **f** Dispersion image derived from the wavefield in panel **d**, revealing multiple guided wave modes. The image is normalized by frequency to enhance visibility across the spectrum and is overlaid with dispersion curves obtained from SAFE-based guided wave modeling (black dots; see "Methods" for details). Simulation results show strong agreement with the observed dispersion patterns below 100 Hz, particularly for the fundamental symmetric (S_0) and antisymmetric (A_0) modes. Blue and green triangles in panel **f** indicate the modes shown in panels **g-h**, respectively. **g** Out-of-plane displacement of the A_0 mode at 40 Hz (phase velocity 1728 m/s), antisymmetric about the central dashed line. **h** Out-of-plane displacement of the S_0 mode at 50 Hz (phase velocity 3902 m/s), symmetric about the central dashed line.

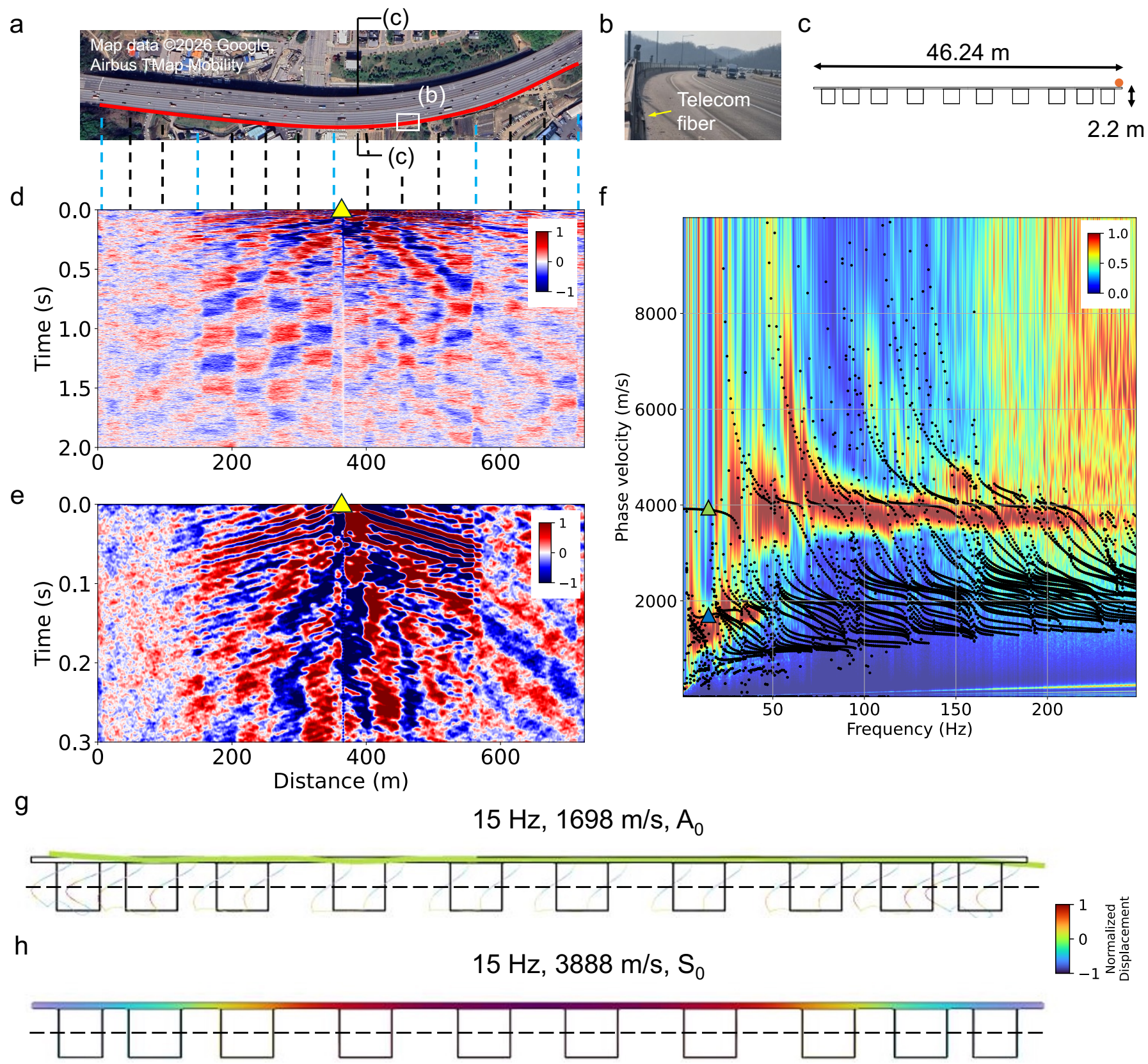

Supplementary Fig. 4: **Analysis of guided wavefields in the Bakdal 2 bridge using Telecom as Sensors.** **a** Aerial image of the bridge from Google Earth, with the red line indicating the route of the pre-existing telecom fiber-optic cable. Vertical dashed lines denote the locations of bridge piers (black) and expansion joints (blue). **b** Photo showing the side of the bridge where the telecom cable is laid along the edge of the top surface. **c** Cross-section of the bridge. The orange dot indicates the location of the mounted fiber. **d** Virtual source gather showing the propagation of guided waves along the bridge. The data are normalized for visualization. The yellow triangle marks the virtual source location. **e** Zoomed-in view of panel **d** from 0 to 0.3 seconds, showing dispersive higher-frequency guided waves with faster propagation speeds. **f** Dispersion image derived from the wavefield in panel **d**, revealing multiple guided wave modes. The image is normalized by frequency to enhance visibility across the spectrum and is overlaid with dispersion curves obtained from SAFE-based guided wave modeling (black dots; see "Methods" for details). Blue and green triangles in panel **f** indicate the modes shown in panels **g-h**, respectively. **g** Out-of-plane displacement of the A_0 mode at 15 Hz (phase velocity 1698 m/s), antisymmetric about the central dashed line. **h** Out-of-plane displacement of the S_0 mode at 15 Hz (phase velocity 3888 m/s), symmetric about the central dashed line.

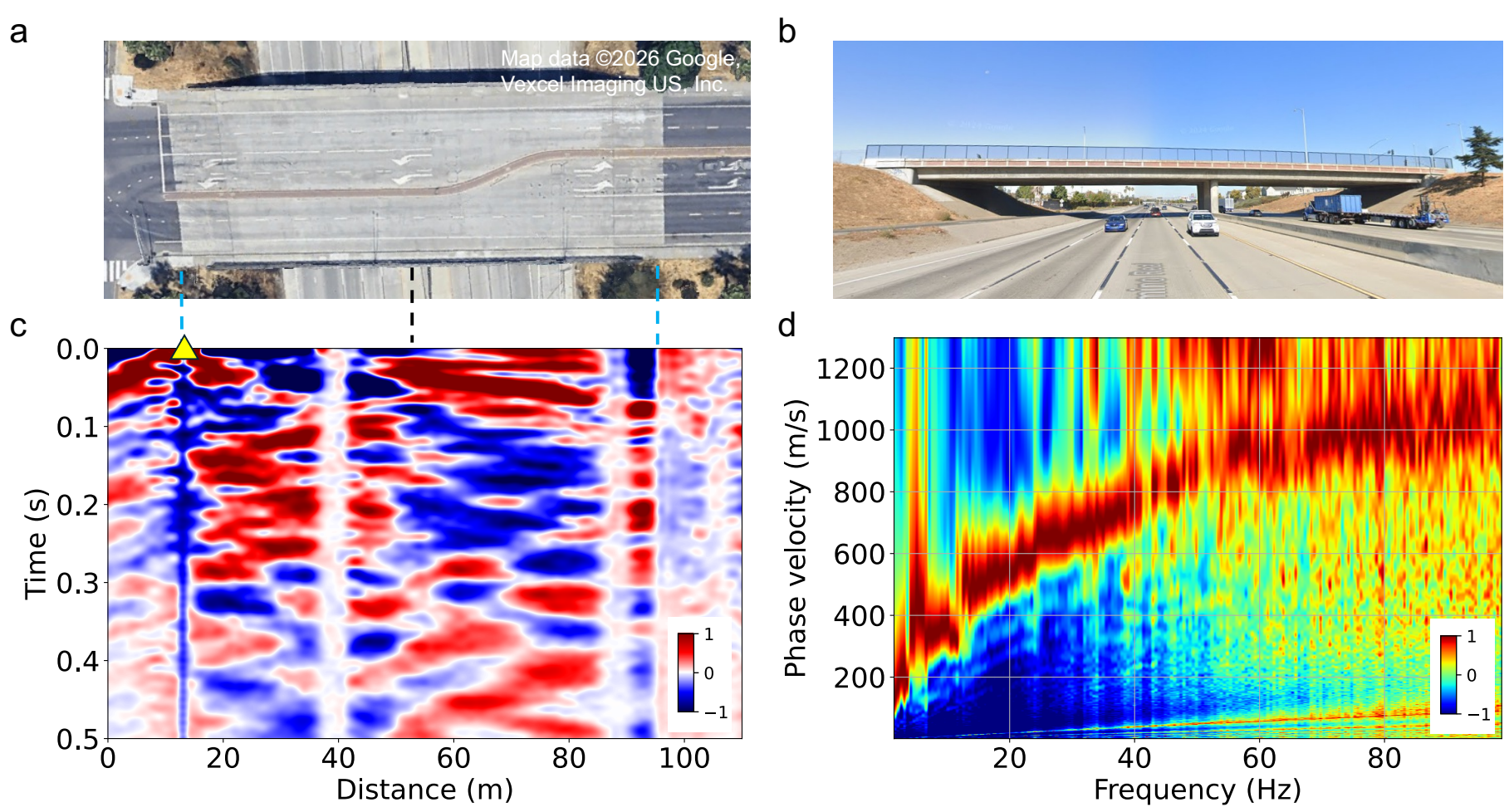

Supplementary Fig. 5: **Analysis of guided wavefields in the McKee Road overpass using Telecom as Sensors.** **a**–**b**, Aerial and side views of the bridge from Google Earth. The location and coupling method of the telecom cable at the McKee Road overpass were not accessible and could not be visually confirmed. **c**, Virtual source gather showing guided wave propagation along the bridge. The data are normalized for visualization, and the yellow triangle marks the virtual source location. **d**, Dispersion image derived from the wavefield in panel **c**. Due to the 200 Hz sampling rate of the DAS system, only the fundamental antisymmetric mode (A_0) is observed. SAFE-based guided wave modeling was not conducted due to the unavailability of cross-sectional design drawings for the bridge.

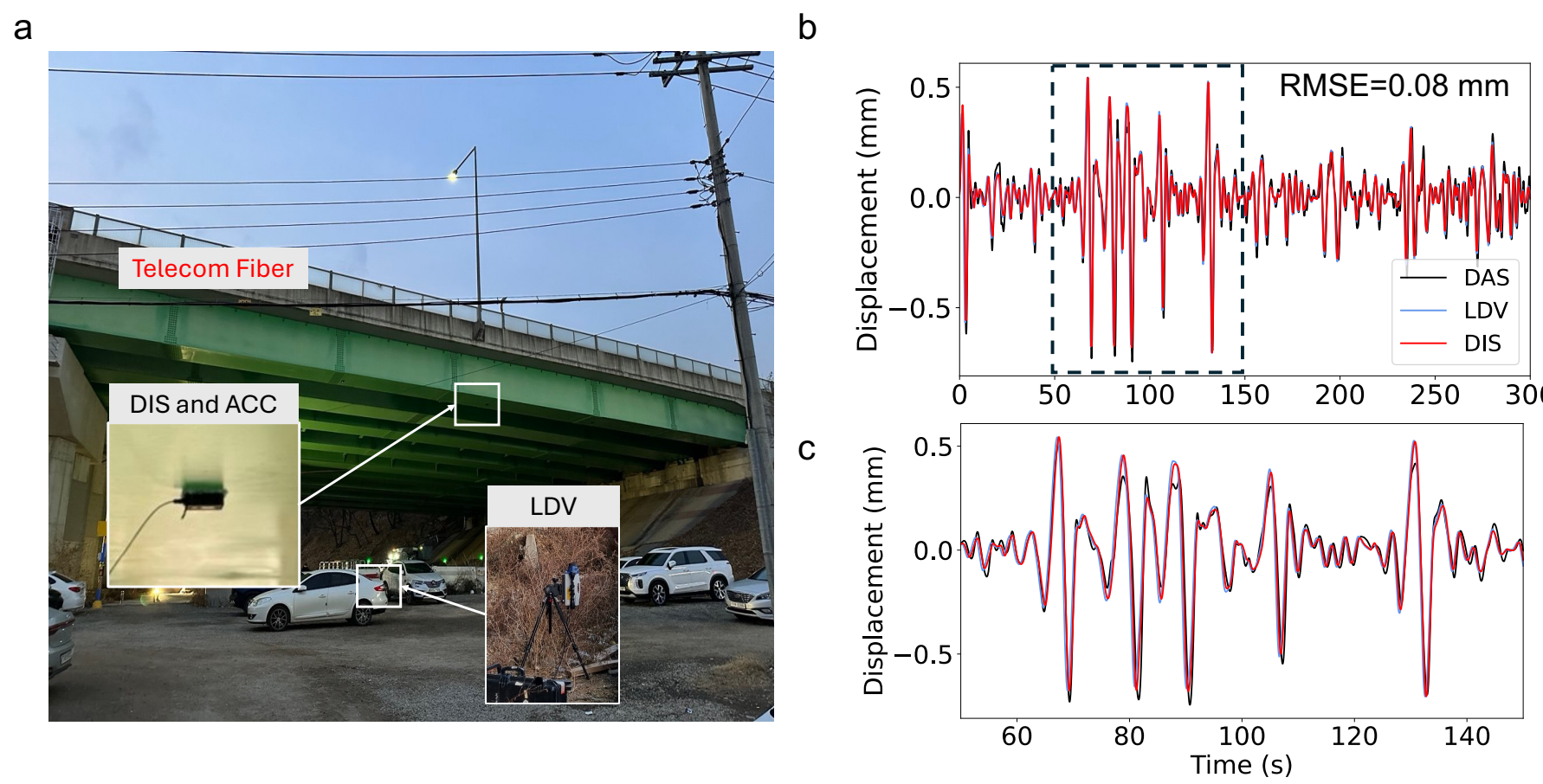

Supplementary Fig. 6: **Quasi-static displacement estimation for the Bakdal 2 bridge using Telecom as Sensors, validated against conventional sensors.** **a** Field deployment showing the pre-existing telecom fiber-optic cable used for DAS, alongside an accelerometer (ACC), displacement sensor (DIS), and laser Doppler vibrometer (LDV) for ground-truth validation. Insets show close-ups of the LDV and DIS setups. **b** Comparison of vertical displacement time series over a 5-minute interval measured by DAS (black), LDV (red), and DIS (blue). DAS-based displacement estimation achieved a root mean squared error of 0.08 mm compared to LDV. **c** Zoomed-in view of the boxed region in (**b**), illustrating sub-millimeter agreement between DAS and validation sensing modalities in capturing quasi-static displacements.

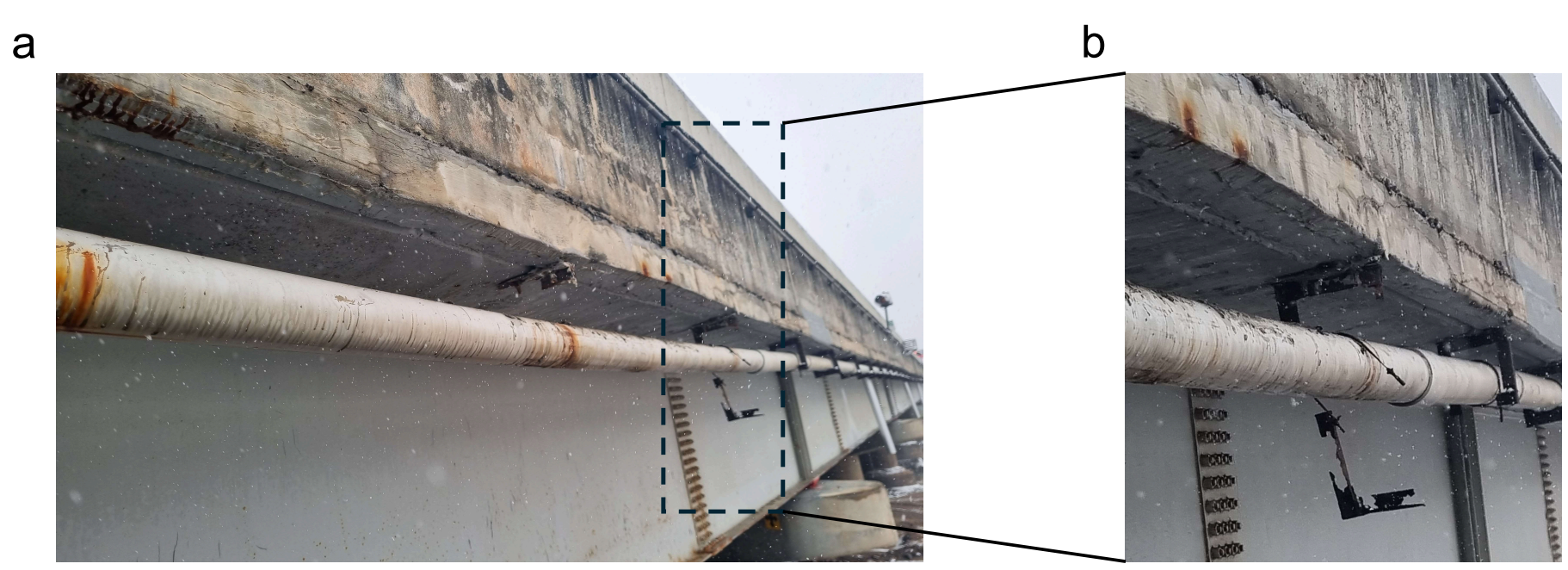

Supplementary Fig. 7: **Photos showing poor coupling of the telecom fiber-optic cable to the Byeongcheon 2 bridge.** **b** is a zoomed-in view of panel **a**. Due to inadequate coupling, reliable quasi-static displacement and modal analysis results could not be obtained.

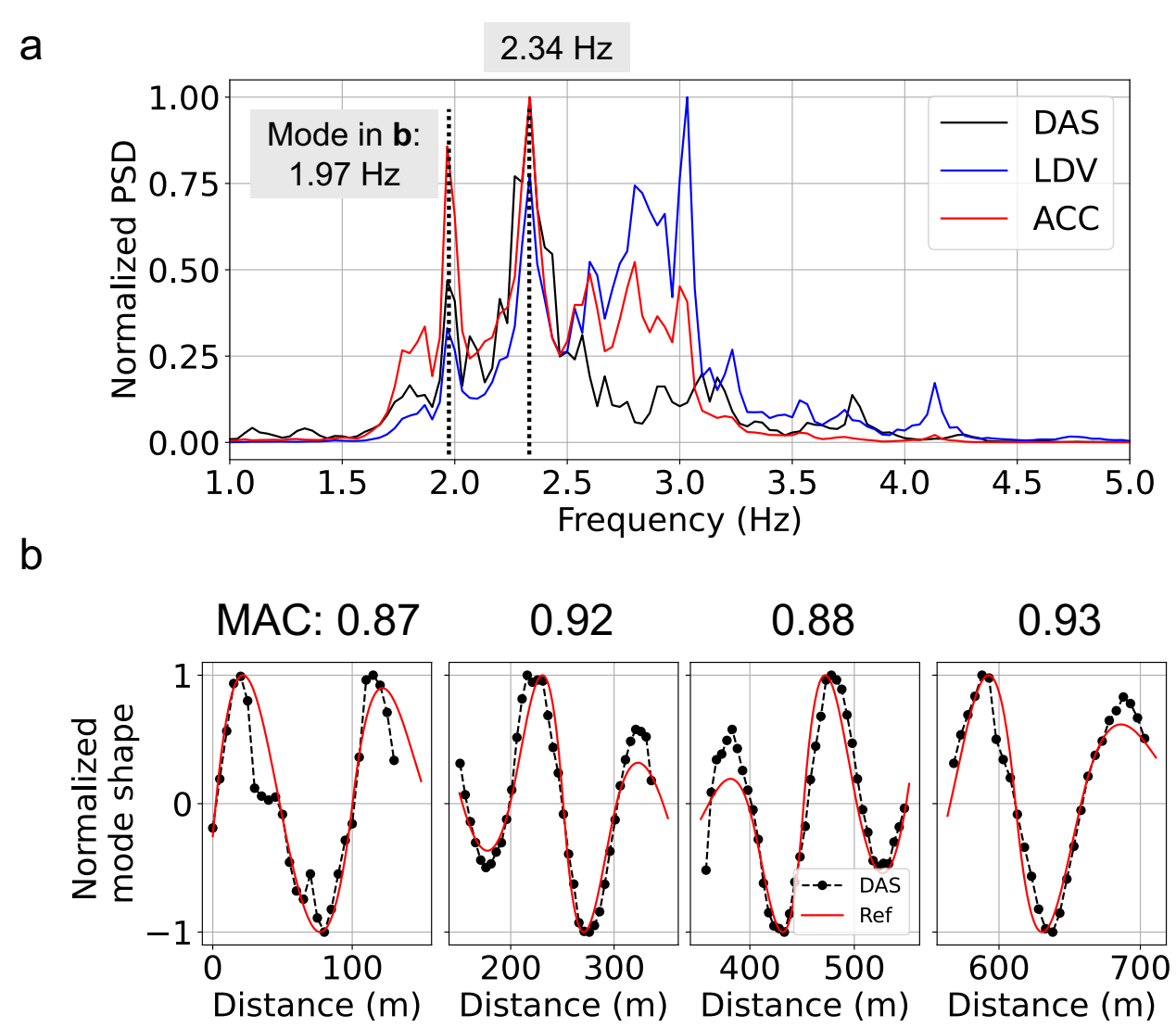

Supplementary Fig. 8: **Frequency response and modal analysis of the Bakdal 2 bridge using Telecom as Sensors, validated against conventional sensors and numerical modeling. a** Normalized power spectral density (PSD) of dynamic responses measured by DAS (black), laser Doppler vibrometer (LDV, blue), and accelerometer (ACC, red), showing consistent identification of the first two modes at 1.97 and 2.34 Hz. **b** Normalized mode shapes corresponding to the identified first mode of the four structural partitions, derived from the DAS response using the Frequency Domain Decomposition (FDD) method (see "Methods" for details) and compared with numerical results from OpenSees. The first mode of the four structural partitions achieves Modal Assurance Criterion (MAC) values of 0.87, 0.92, 0.88, and 0.93, respectively.

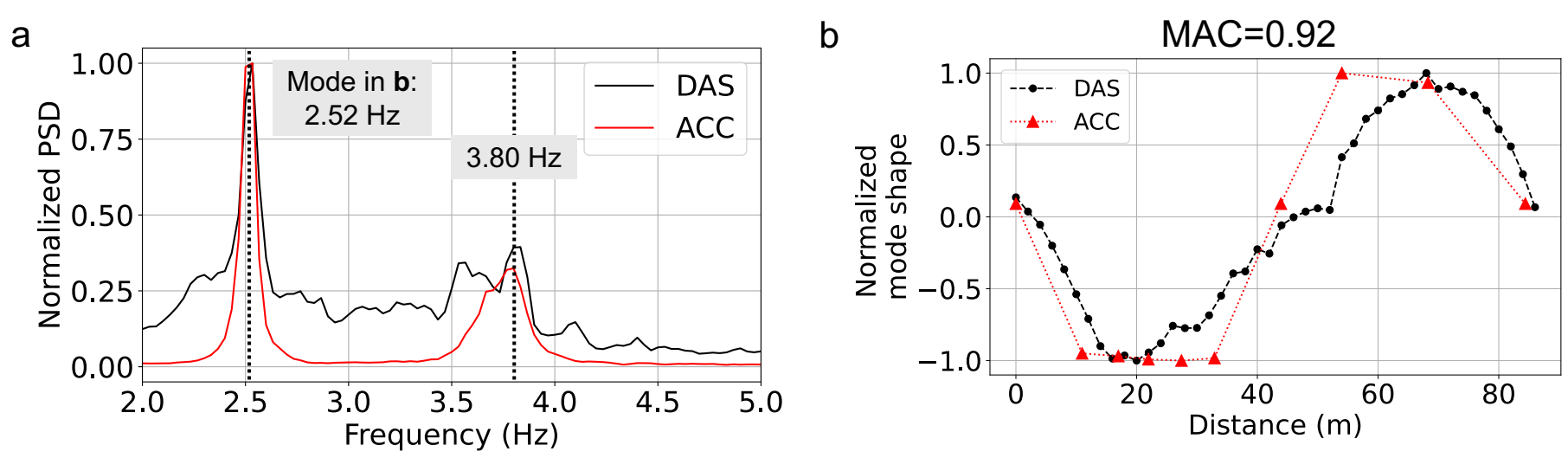

Supplementary Fig. 9: **Frequency response and modal analysis of the McKee Road bridge using Telecom as Sensors, validated against conventional sensors.** **a** Normalized power spectral density (PSD) of dynamic responses measured by DAS (black) and accelerometers (ACC; red), showing consistent identification of the first two modes at 2.52 and 3.80 Hz. **b** Normalized mode shape corresponding to the first mode, derived from DAS data using the Frequency Domain Decomposition (FDD) method (see "Methods" for details) and compared with that derived from accelerometer measurements. Red triangles indicate accelerometer locations. A Modal Assurance Criterion (MAC) value of 0.92 for the first mode confirms strong agreement.

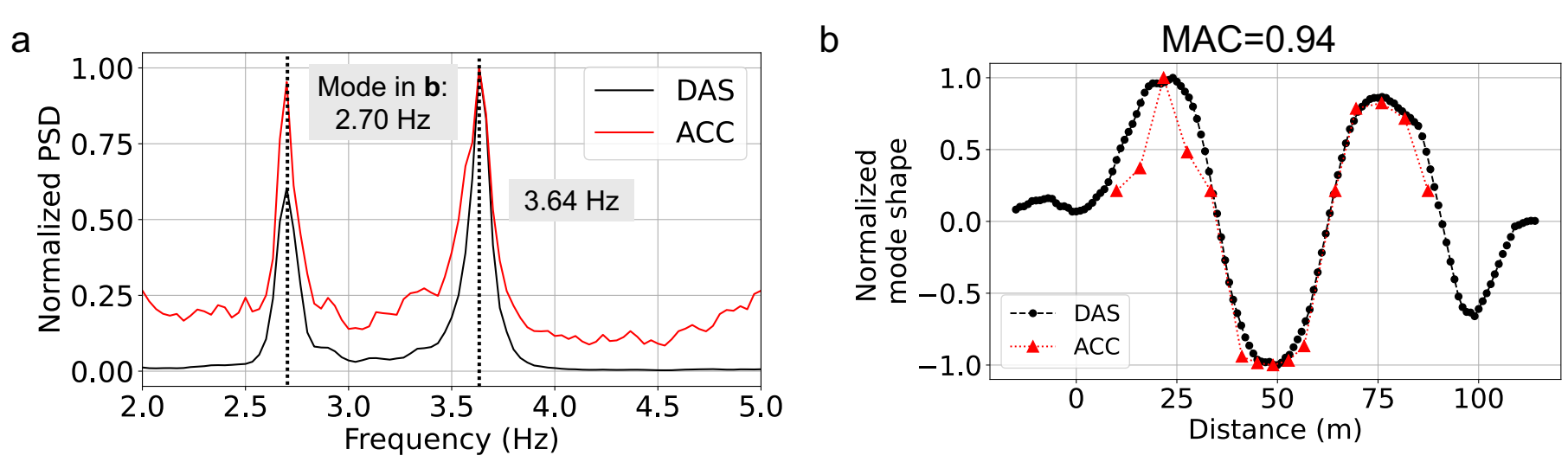

Supplementary Fig. 10: **Frequency response and modal analysis of the Yerba Buena Road bridge using Telecom as Sensors, validated against conventional sensors.** **a** Normalized power spectral density (PSD) of dynamic responses measured by DAS (black) and accelerometers (ACC; red), showing consistent identification of the first two modes at 2.70 and 3.64 Hz. **b** Normalized mode shape corresponding to the first mode, derived from DAS data using the Frequency Domain Decomposition (FDD) method (see "Methods" for details) and compared with that derived from accelerometer measurements. Red triangles indicate accelerometer locations. A Modal Assurance Criterion (MAC) value of 0.94 for the first mode confirms strong agreement.

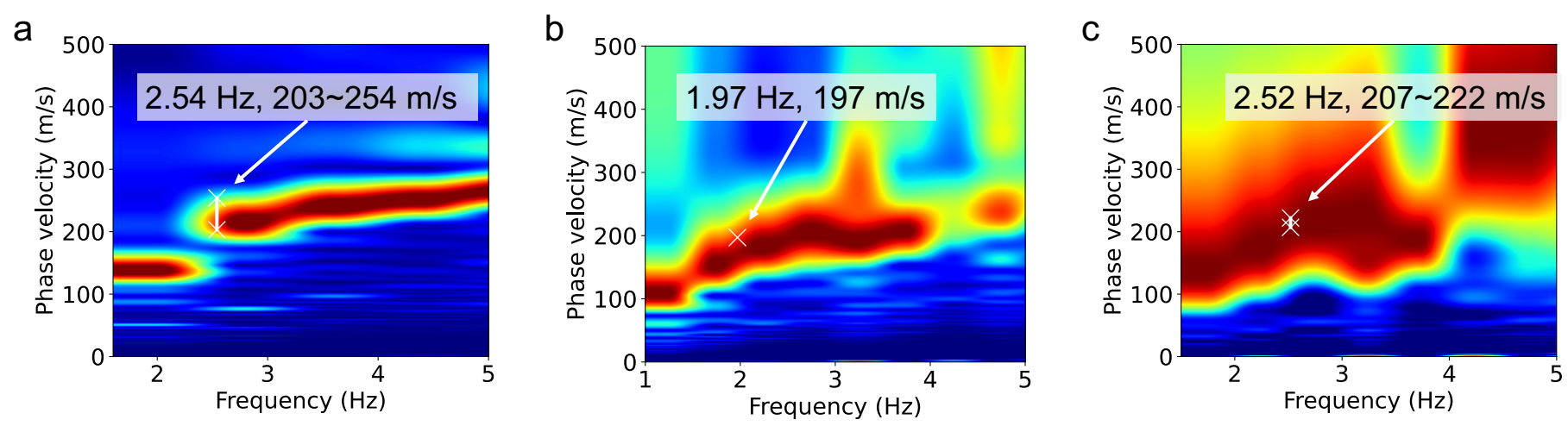

Supplementary Fig. 11: **Resonance frequencies and standing-wave behavior in three bridges.** Measured dispersion spectra below 5 Hz for (**a**) Miho River bridge, (**b**) Bakdal 2 bridge, and (**c**) McKee Road bridge. White crosses and lines indicate the identified resonance frequencies and corresponding standing-wave velocity ranges: 2.54 Hz, 203∼254 m/s (Miho River); 1.97 Hz, 197 m/s (Bakdal 2); and 2.52 Hz, 207∼222 m/s (McKee Road). The close match between modeled and measured signals demonstrates that standing waves arise from the superposition of left- and right-propagating guided waves.

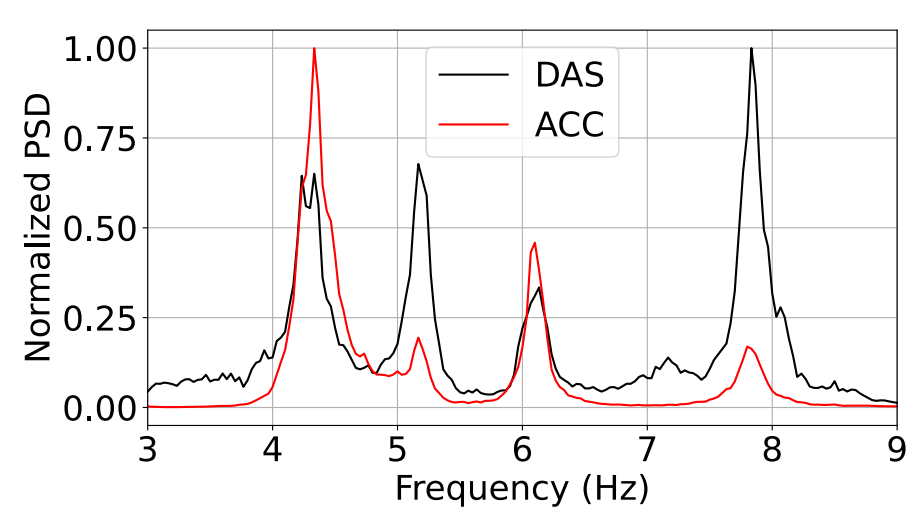

Supplementary Fig. 12: **Normalized power spectral density (PSD) of dynamic responses measured by DAS (black) and accelerometers (ACC; red), showing consistent identification of dominant frequency content.** Reliable mode shape estimates could not be obtained because the bridge length (29.3 meters) is too short relative to the DAS gauge length (16.3 meters), limiting spatial resolution.

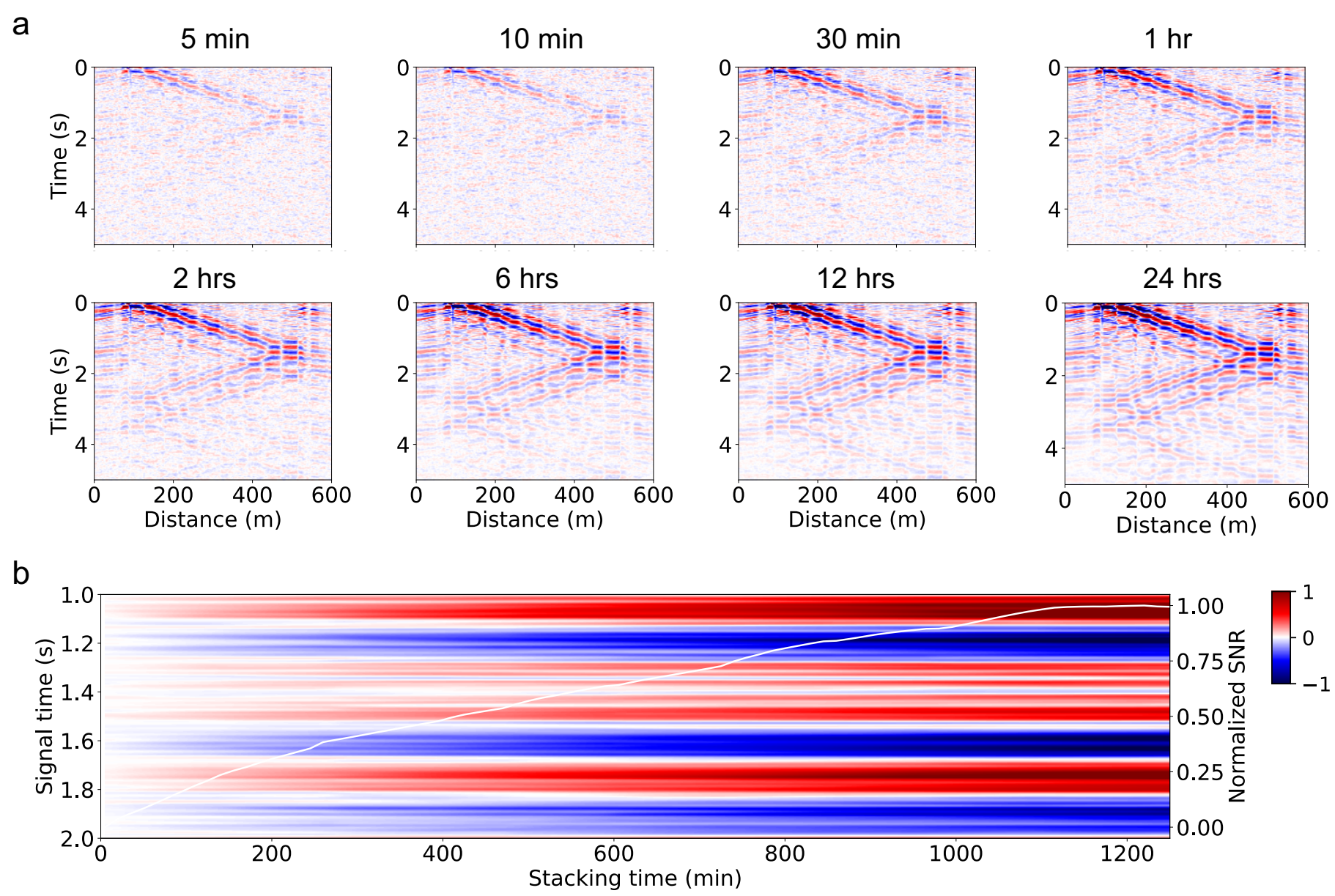

Supplementary Fig. 13: **Influence of stacking duration on the retrieval of guided waves from traffic-induced ambient noise recorded by DAS.** **a** Virtual Source Gathers (VSGs) obtained via ambient noise interferometry using stacking durations ranging from 5 minutes to 24 hours. A single DAS channel near the left expansion joint is used as the virtual source. Coherent guided wave modes become increasingly visible with longer stacking times, exhibiting stronger signal amplitude and improved continuity across the array. **b** Signal-to-noise ratio (SNR) evolution of a single VSG trace at 300 m offset as a function of stacking duration. SNR is calculated as the energy ratio between a signal window (1–2 seconds) and a subsequent noise window. Results show a near-linear SNR increase with stacking time, with convergence reached after approximately 20 hours.
