# Supplementary material for: Bridge health monitoring using existing telecommunication fiber-optic networks
Source: Nat Commun. 2026 Aug 21;17:9966. doi: 10.1038/s41467-026-76935-0 (PMC13586185; doi:10.1038/s41467-026-76935-0)
Supplement: Supplementary file 2 [file 41467_2026_76935_MOESM2_ESM.pdf]

nature portfolio

Peer Review File

Bridge Health Monitoring Using Existing Telecommunication Fiber-Optic Networks

Corresponding Author: Dr Jingxiao Liu

This file contains all reviewer reports in order by version, followed by all author rebuttals in order by version.

Version 0:

Reviewer comments:

Reviewer #1

(Remarks to the Author)
Solid work with strong methodology and interpretations. As the DAS research community moves away from costly deployments of dedicated fiber, this study presents useful findings for leveraging "Telecom as Sensors". I appreciate the presentation of results from six different bridges and the demonstration of the applicability of the authors' methods across these different settings. I also appreciate the detailed validation of results (e.g., the use of SAFE for validating interpretations of dispersion images), which I believe is of higher quality than many existing DAS papers. Detailed descriptions of deployed fibers and a strong theoretical grounding are a plus. Some figures could be improved, such as adding further annotations of the relevant details (e.g., Figure 1.c components of the strain signatures could be annotated), but this does not impact my recommendation of this paper for publication.

(Remarks on code availability)
I did not download and run the code myself, but I reviewed the scripts and functions for generating the figures, which seem solid. The code does not provide a README file, howevever, which should be included.

Reviewer #2

(Remarks to the Author)
General Assessment
This manuscript presents a very interesting and timely study that combines multiple data types to demonstrate the relevance of an innovative bridge monitoring approach based on telecom optical fibers and Distributed Acoustic Sensing (DAS). The joint analysis of modal properties and guided wave propagation is particularly valuable and places the work at the frontier of large-scale structural health monitoring using existing telecom infrastructure.
The paper is well written, the experimental work is convincing, and the methodology is generally sound. The study clearly has the potential to make a meaningful contribution to the field. The following comments mainly aim at clarifying the positioning of the work with respect to existing literature, strengthening the interpretation of the results, and improving methodological transparency. These points can be addressed without major additional experiments.

Noteworthy Results and Originality
The most noteworthy contributions of this study are:
• The monitoring of several bridges along a single telecom fiber,
• The displacement-based interpretation of DAS measurements,
• The investigation of guided wave (Lamb wave) diffraction phenomena along bridge structures.
It has already been demonstrated that telecom fiber networks can be used to perform modal analysis of both concrete and steel bridges (Rodet, J., Tauzin, B., Amin Panah, M., Guéguen, P., Nziengui Bâ, D., Coutant, O., & Brûlé, S. (2025). Urban dark fiber distributed acoustic sensing for bridge monitoring. Structural Health Monitoring, 24(1), 636-653.). Therefore, the modal analysis itself should not be presented as a fundamentally new contribution. Some statements in the manuscript (notably around lines 376–378) should be slightly revised to better acknowledge this prior work and to clearly distinguish what has already been demonstrated from what is newly proposed here.
The originality of the manuscript lies more convincingly in the extension beyond classical modal analysis, especially through displacement-based interpretation and guided wave field analysis. Repositioning the novelty around these aspects would strengthen the manuscript.

Significance to the Field and Relation to the Literature
The work is of clear interest for the structural health monitoring and civil engineering communities. The use of existing telecom fibers for large-scale and continuous monitoring remains highly attractive, and this study further supports its feasibility.
To further improve the manuscript, the comparison with existing literature could be slightly expanded, particularly by:
• More explicitly referencing previous modal analysis studies using DAS,
• Clarifying how the present work extends these studies, especially regarding guided wave observations and interpretation.

Methodology and Reproducibility
The methodology is clearly described and generally meets the expected standards of the field. A few clarifications would nevertheless improve reproducibility and interpretation:
• Coupling effects (lines 385 and 433):
A short discussion on how coupling between the fiber and the structure may affect the DAS measurements would be beneficial.
• Blind applicability of the method:
It would be useful to discuss whether this approach could be applied without numerical models or reference measurements, or whether it should preferably be combined with more conventional monitoring approaches.
• Guided waves and damage detection (line 392):
The manuscript would benefit from a clearer explanation of how guided wave measurements could be exploited for damage detection and how damage-related effects could be distinguished from coupling or environmental effects.
• Cost statement (line 394):
Emphasizing the ease of deployment is appropriate, but the statement regarding low cost could be slightly nuanced given the price of DAS interrogators.
• DAS interrogator characteristics (line 434):
Please specify the type of interrogator used (e.g., single-pulse or dual-pulse) and briefly discuss whether the results could differ between interrogators.
• Noise level and spatial sampling (line 455):
A short discussion on the noise level in the data and on the choice of spatial sampling and gauge length would strengthen the methodological section.
• Sampling frequency (line 460):
The impact of the sampling frequency on the analysis could be briefly discussed, especially given the low-frequency content of interest.
• Strain vs. strain-rate (line 462):
Please justify the choice of strain measurements instead of strain-rate, which is often better suited for low-frequency applications and avoids phase unwrapping issues.
• Removal of low-order modes (line 512):
A brief discussion on the implications of removing the first modes for bridge monitoring would be helpful.
• Integration method (line 605):
Please clarify whether the chosen integration method may influence the results.
• Operational modal analysis assumptions (lines 615–617):
It would be helpful to recall the fundamental assumptions of operational modal analysis under ambient excitation.
• Spatial resolution (lines 634–635):
Since spatial resolution depends on gauge length, please clarify that modal shape resolution may differ between bridges.

Interpretation and Conclusions
The conclusions are generally supported by the results. The section on guided wave analysis (lines 503–508) is particularly interesting and appears to go beyond what has been previously demonstrated. A slightly more explicit discussion on how these observations could be used for structural damage detection would further strengthen the manuscript.

Recommendation
In conclusion, this manuscript presents a solid and interesting contribution to the field. The comments above mainly concern clarification, positioning with respect to existing literature, and minor methodological discussions. They can be addressed without major additional work.
I therefore recommend publication after minor revision.

(Remarks on code availability)
To facilitate the reuse of the provided code by the community, I strongly encourage the authors to include a clear and comprehensive README file. At present, the structure, dependencies, and execution workflow of the code are not sufficiently documented to allow straightforward reuse by peers. A well-written README should describe the installation procedure, software and library dependencies, input data format, and step-by-step instructions to reproduce the main results presented in the paper. While the availability of the code is a positive aspect and supports transparency, the reproducibility of the results and the usability of the code as a community resource would be significantly improved by clearer documentation.

Reviewer #3

(Remarks to the Author)
General Review:
The paper presents new structural health monitoring paradigm that uses existing fiber-optic telecommunication cables as a scalable and cost-effective approach. Fiber-optic cables are used as arrays of dynamic strain sensors for continuous bridge health monitoring, and quasi-static bridge displacements and dynamic vibrations are obtained. Six real-world case studies are used to demonstrate the applicability of the proposed method and the measurements are verified using LDV and accelerometers, as well as with numerical analysis results.

The current study is on a topic of relevance and general interest to the readers of the journal. Also, the proposed methodology/paragidm is quite interesting and exciting and the paper is written with sufficient quality and detail. However, several issues raised below should be clarified before the publication of the paper.

The authors could benefit from the comments below to improve their paper.
Major Comments:
- Introduction. The novelty of the study should be highlighted better by i) referring to more recent literature (especially for the review papers used, there are much more recent review papers), ii) stressing the efforts to overcome the problems associated with SHM in the last decades (see review paper by Farrar et al., 2025), iii) distinguishing this work from the earlier works of the authors clearly and ensuring that it is not incremental.
See for example:
Liu et al.- Turning Telecommunication Fiber-Optic Cables into Distributed Acoustic Sensors for Vibration-Based Bridge Health Monitoring, https://doi.org/10.1155/2023/3902306
Liu et al.- Urban sensing using existing fiber-optic networks. https://doi.org/10.1038/s41467-025-57997-y
Donadello, S., Clivati, C., Govoni, A. et al. Seismic monitoring using the telecom fiber network. Commun Earth Environ 5, 178 (2024). https://doi.org/10.1038/s43247-024-01338-2

- Introduction. The claims of “maintenance free” and “highly accurate” should be explained further.
- Fig 1. According to my understanding, the time-history of DAS presented in Fig (b), whose magnitude is between 0.4-0.6, seems to differ from the plot presented in Fig (c). For the location of the sensor (b), the spectrum in Fig (c) changes from negative to positive values (blue to red) – different from the values of the Fig (b), even though the location of the peak seems correct. Please explain/revise.
- Methodology. It is not clear how the Telecom as Sensors are attached to the bridge structures. Is the same method applied for all of them? What are the implications of the type of the attachment and its location on the bridge? Figure 3b shows that it passes through a metal tube that is clamped to the flange of the bridge whereas other types of attachments are observed as well.
- Figure 3g-h. I do not understand the explanation in the Results section. It is very unlikely that the first bridge modes are at 40-50Hz.
- Sandhill Road bridge and Yerba Buena Road bridge examples show the limitations of the proposed method, that are the structural and sensing contraints mentioned. It might be useful to summarize those in the discussion section.
- Methodology. Do Telecom as Sensors measure in the longitudinal direction of the cables only, in-line with the strains/strain rates measurements or in three orthogonal directions as well? Can you please clarify?
- Methodology. Description of the monitored bridges and Fig 2 seem insufficient in terms of details. Although these bridges and structural features/behavior are integral to the study, the presented information gives the impression that they are complementary. I recommend providing more details and figures (if possible).
- Methodology. DAS data acquisition. Please provide more information on how telecom optical fibers system function, i.e., operated by whom, where the interrogators are installed, their properties, if the data is publicly available, and more practical data to enable others to use the same methodology.
- Methodology. Retrieving guided wavefields from DAS data. What are the practical implications of reaching the SNR convergence after approximately 20 hours? Please explain.
- Methodology. A figure or a chart explaining the methodology step-by-step would be useful; otherwise, it is difficult to follow for someone like me who is not an expert on guided waves. For example, once the guided wave fields and VSGs are obtained, what can they be used for? Then, dispersion images are obtained from VSGs, and what are they used for? Only for checking the dispersion curves obtained from SAFE-based guided wave modeling? The numerical modeling serves to find the mode shapes only? Have you provided all the necessary details to model these six bridges for the repeatability of the study? Please clarify.
- Methodology. Modal analysis using FDD and the related figures. The details of the analysis must be provided, i.e., length of the signal, number of fft used in the transformation, etc. both for the DAS and ACC. It seems that the signal durations for accelerometers are quite short as the resulting spectra are very coarse. If that is the case, why not use longer data? Please explain and justify.
- Methodology. Modal analysis using FDD. How do the modes presented in Figures 3 and 5 compare? Are they related?
- Methodology. Numerical modeling of bridge mode shapes. Employed modeling approach with Bernoulli-Euler beams does not permit distinguishing torsional modes or transverse vs. vertical modes. While the Telecom as Sensors pass through one side of the bridge (as commonly seen in the study), the same side will show modal displacements for transverse and torsional modes in addition to the vertical modes. How do you distinguish them? If you had a single span bridge, then the first transverse and vertical modes would look the same (considering the same measurement configurations). Could you please explain and clarify?

(Remarks on code availability)

Version 1:

Reviewer comments:

Reviewer #1

(Remarks to the Author)
Thank you for addressing the comments. Responses to the other reviewers seem solid as well.

(Remarks on code availability)

Reviewer #2

(Remarks to the Author)
Dear Authors,

Thank you for your detailed and thoughtful revision of the manuscript.

I appreciate the considerable effort made to address all reviewers' comments. The revised manuscript is substantially improved, especially regarding the clarification of the scientific novelty, the discussion of practical limitations and coupling conditions, and the additional methodological details provided throughout the paper.

The new explanations concerning guided-wave interpretation, operational constraints, interrogator configurations, and the relationship between modal analysis and guided-wave behavior greatly strengthen the manuscript. The addition of the methodological flowchart and the expanded discussion sections also improve the readability and accessibility of the work.

Overall, this is a very strong, complete, and highly interesting paper for the advancement of DAS-based Structural Health Monitoring. I believe it will be of significant interest to the SHM and fiber-optic sensing communities.

Congratulations on this excellent work.

Best regards

(Remarks on code availability)

Reviewer #3

(Remarks to the Author)
I thank the authors for their diligent work in responding the reviewers' comments. The article has improved significantly and I hope it helps advancing our field and the science.

(Remarks on code availability)

Open Access This Peer Review File is licensed under a Creative Commons Attribution 4.0 International License, which permits use, sharing, adaptation, distribution and reproduction in any medium or format, as long as you give appropriate credit to the original author(s) and the source, provide a link to the Creative Commons license, and indicate if changes were made.
In cases where reviewers are anonymous, credit should be given to 'Anonymous Referee' and the source.
The images or other third party material in this Peer Review File are included in the article's Creative Commons license, unless indicated otherwise in a credit line to the material. If material is not included in the article's Creative Commons license and your intended use is not permitted by statutory regulation or exceeds the permitted use, you will need to obtain permission directly from the copyright holder.
To view a copy of this license, visit https://creativecommons.org/licenses/by/4.0/

Point-by-point response:

We would like to thank the reviewers for their thoughtful comments and constructive suggestions, which have helped to improve the manuscript.

We have made modifications to the manuscript to address all reviewers' comments. Below is the list of each of the reviewers' comments (in blue), followed by our corresponding responses and the updated text in the manuscript (in red).

Response to Reviewer 1:

Comment:
Solid work with strong methodology and interpretations. As the DAS research community moves away from costly deployments of dedicated fiber, this study presents useful findings for leveraging "Telecom as Sensors". I appreciate the presentation of results from six different bridges and the demonstration of the applicability of the authors' methods across these different settings. I also appreciate the detailed validation of results (e.g., the use of SAFE for validating interpretations of dispersion images), which I believe is of higher quality than many existing DAS papers. Detailed descriptions of deployed fibers and a strong theoretical grounding are a plus. Some figures could be improved, such as adding further annotations of the relevant details (e.g., Figure 1.c components of the strain signatures could be annotated), but this does not impact my recommendation of this paper for publication.

Response:
We thank Reviewer #1 for the positive assessment of our methodology, validation, and interpretations, and for the constructive suggestion regarding figure clarity. In response, we revised Figure 1c to include additional annotations that label the key components of the spatiotemporal strain signatures, such as the trajectory of the passing vehicle and the polarity reversals associated with piers and joints, so the relevant features are easier to identify.

Revised manuscript:
The updated Figure 1 (including the revised Figure 1c) is attached below.

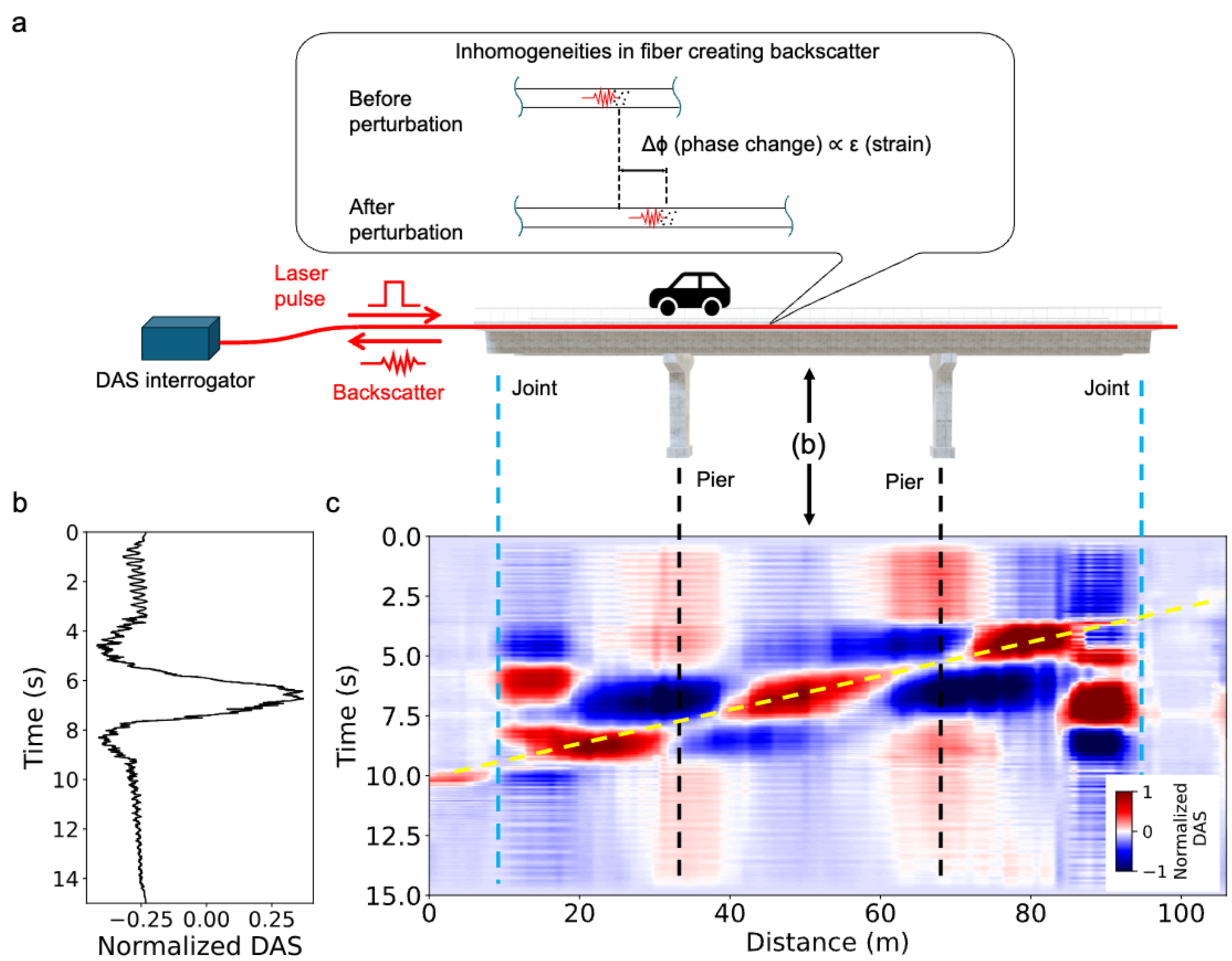

Figure 1. Monitoring bridge responses using Telecom as Sensors. **a** Schematic of the DAS measurement principle. Laser pulses are injected into a telecom fiber coupled to a three-span bridge. Microscopic inhomogeneities in the fiber cause Rayleigh backscattering. External strain, such as that induced by a passing vehicle, alters the optical path length, producing a phase shift ($\Delta\phi$) in the backscattered signal proportional to the axial strain ϵ. **b** Time series of the DAS response recorded at the mid-span (location marked by the double-headed arrow in panels **a** and **c**), capturing vehicle-induced strain as the vehicle travels from right to left. Both dynamic and quasi-static components of the response are observed. **c** Space-time plot of normalized DAS response along the full bridge span. The vehicle's passage from right to left produces clear spatiotemporal strain signatures. The yellow dashed line denotes the vehicle trajectory. Vertical dashed lines denote expansion joints and pier locations.

Comment (Remarks on code availability):
I did not download and run the code myself, but I reviewed the scripts and functions for generating the figures, which seem solid. The code does not provide a README file, however, which should be included.

Response:
We thank the reviewer for examining the code. We have added a comprehensive README file to the code repository at https://github.com/jingxiaoliu/das_bhm.

Response to Reviewer 2:

Comment:
It has already been demonstrated that telecom fiber networks can be used to perform modal analysis of both concrete and steel bridges (Rodet, J., et al. (2025). Urban dark fiber distributed acoustic sensing for bridge monitoring. Structural Health Monitoring, 24(1), 636-653.). Therefore, the modal analysis itself should not be presented as a fundamentally new contribution. Some statements in the manuscript (notably around lines 376–378) should be slightly revised to better acknowledge this prior work and to clearly distinguish what has already been demonstrated from what is newly proposed here. The originality of the manuscript lies more convincingly in the extension beyond classical modal analysis, especially through displacement-based interpretation and guided wave field analysis. Repositioning the novelty around these aspects would strengthen the manuscript.
To further improve the manuscript, the comparison with existing literature could be slightly expanded, particularly by: More explicitly referencing previous modal analysis studies using DAS; Clarifying how the present work extends these studies, especially regarding guided wave observations and interpretation.

Response:
We thank Reviewer #2 for highlighting the relevant prior work and for the helpful suggestion to more clearly distinguish established capabilities from the novel contributions of this study. We would like to note that our earlier work (Liu et al., 2023), as well as Rodet et al. (2025), showed the demonstration of bridge modal analysis using urban dark fiber DAS. At the same time, we agree that telecom and dark fiber DAS-based modal analysis has now been demonstrated in the literature, and we have revised the manuscript to acknowledge this more explicitly and to better position the novelty of the present study accordingly.

Specifically, we have (i) expanded the comparison with prior literature by explicitly citing both Liu et al. (2023) and Rodet et al. (2025) in the context of telecom-fiber DAS modal identification, and (ii) revised the corresponding discussion in the revised manuscript (L400–407) to clarify that the primary originality of the present manuscript lies beyond classical modal analysis. In particular, we emphasize the extensions to displacement-based interpretation and guided-wave field retrieval and analysis, and we clarify how these components build on and extend prior modal-analysis-focused studies.

Revised manuscript:

Analysis of dynamic responses, L289–302: “DAS using telecom fiber-optic cables coupled to bridges also enables reliable measurement of bridge dynamic responses. Liu et al. [28] first performed modal analysis using Telecom as Sensors on a continuous three-span concrete bridge,

successfully estimating resonant frequencies and mode shapes. Recently, Rodet et al. [29] further evaluated the feasibility of using dark fiber to monitor multiple bridges across an extended fiber network and captured daily variations in modal parameters using DAS on dark fiber… In this study, we extend the validation of Telecom as Sensors for dynamic response monitoring to additional bridges with diverse structural types (box girders and composite concrete–steel I-beams), span configurations, and lengths."

Discussion section, L404–412: "Beyond cost efficiency, the high spatiotemporal resolution of DAS provides unique measurement capabilities. It enables meter-scale resolution of bridge mode shapes, demonstrating that telecom fiber networks can be used for modal analysis, and it further enables continuous monitoring of guided wavefields along the bridge superstructure and quasi-static displacement estimation from the same sensing system. This combination of capabilities, together with validation across six bridges and year-long monitoring, distinguishes the present work from earlier telecom-fiber studies."

Comment:
A short discussion on how coupling between the fiber and the structure may affect the DAS measurements would be beneficial.

Response:
We agree that coupling is an important factor and appreciate this suggestion. The revised manuscript now addresses this issue in multiple places. In the Abstract (L36–37), we state that the approach is robust to noise from imperfect fiber–bridge coupling. In the Results section, we document how poor coupling at the Byeongcheon 2 bridge limited quasi-static displacement estimation (L280–281) and degraded dynamic-response measurements (L299–301), with photographic evidence in Supplementary Fig. 3. In the Discussion (L409–412), we further note that cable-to-bridge coupling strength is one of the key factors influencing the reliability of DAS-based measurements.

To further address this point, we have added a dedicated paragraph in the Methods section discussing coupling mechanisms and their potential effects on measurements. Specifically, the telecom cables are housed in air-filled PVC conduits and secured to the bridge with metal clamps (Figs. 3b, Extended Data Figs. 1b, 2b). This indirect coupling through the conduit introduces mechanical filtering that can attenuate high-frequency signals and reduce strain transfer efficiency compared to directly bonded fibers. The Byeongcheon 2 bridge case (Supplementary Fig. 3) illustrates how inadequate coupling can preclude reliable quasi-static and modal analysis, while the successful results on the remaining five bridges demonstrate that the clamp-based coupling, when reasonably tight, is sufficient for the analyses presented.

Revised manuscript:

Methods, DAS data acquisition, L506–518:
"The quality and interpretability of DAS measurements depend on the mechanical coupling between the telecom cable and the bridge, which governs strain transfer from the structure to the fiber. Because the fiber is coupled through a conduit that is mechanically clamped to the structure, rather than being directly bonded to the bridge, spatial variability in clamp tightness and conduit contact can produce along-cable variations in overall measurement quality. The Byeongcheon 2 bridge illustrates that inadequate coupling (Supplementary Fig. 3) can limit reliable recovery of quasi-static displacement and degrade modal analysis performance. In contrast, the successful results from the other five bridges indicate that clamp-based coupling, when reasonably tight and continuous, is sufficient for the dynamic response, guided-wavefield, and quasi-static displacement analyses presented here."

Comment:
It would be useful to discuss whether this approach could be applied without numerical models or reference measurements, or whether it should preferably be combined with more conventional monitoring approaches.

Response:
The approach can largely be applied without numerical models or permanent reference measurements. Guided wavefield retrieval, resonant-frequency identification, and mode-shape extraction are carried out directly from the DAS data and do not require prior structural information. Quasi-static displacement estimation does require a one-time calibration factor (the scaling factor z in Eq. 4), which can be obtained either from a single reference measurement or from design drawings if the neutral-axis location is known.

That said, in practice, combining telecom-fiber DAS with conventional sensors may still be advantageous for additional validation and for capturing response components that are not accessible to DAS alone, such as transverse vibrations when the fiber is oriented longitudinally. We have added a brief discussion of this blind-applicability aspect to the Discussion section.

Revised manuscript:
Discussion, L365–375: "Notably, many of the analyses presented here, including guided wavefield retrieval, resonant frequency identification, and mode shape extraction, are performed directly from the DAS data without requiring numerical models or reference sensors. In this study, the numerical models and conventional sensors were used exclusively for validation. However, absolute displacement estimation requires a one-time calibration based on either a reference measurement or design drawings, provided that the neutral axis location is known. In practice, combining Telecom as Sensors with a limited number of conventional instruments could further improve validation and interpretation, particularly for capturing response components that are not aligned with the fiber axis."

Comment:
The manuscript would benefit from a clearer explanation of how guided wave measurements could be exploited for damage detection and how damage-related effects could be distinguished from coupling or environmental effects.

Response:
We appreciate this suggestion. Guided waves are sensitive to local changes in material properties, geometry, and boundary conditions (L159–160), and the higher-frequency modes are particularly sensitive to local geometric and structural variations (L218–221). In the context of damage detection, localized structural degradation (e.g., cracking, delamination, or section loss) would alter the dispersion characteristics, produce new reflections, or cause mode conversions that would be detectable in repeated measurements over time. We now describe this point more explicitly in the Discussion (L439–448).

To address the question of distinguishing damage from coupling or environmental effects, we have expanded the Discussion to note that environmental effects (e.g., temperature) tend to produce spatially uniform changes in wave propagation properties, whereas localized damage produces spatially concentrated anomalies. Coupling variations would affect signal amplitude but are less likely to alter the dispersion relationship itself. These distinctions provide a pathway for separating damage signatures from confounding factors, though developing robust automated detection remains a direction for future work.

Revised manuscript:
Discussion, L439–448: "Localized structural degradation, such as cracking or section loss, would produce spatially concentrated anomalies in the dispersion characteristics, in contrast to temperature-induced changes, which tend to be spatially uniform; these localized anomalies could then be imaged using guided-wave full-waveform inversion~\cite{10.1190/geo2024-0645.1}."

Comment:
Emphasizing the ease of deployment is appropriate, but the statement regarding low cost could be slightly nuanced given the price of DAS interrogators.

Response:
We agree that the cost of DAS interrogators is a relevant consideration and should be acknowledged. The revised Discussion now states more explicitly that, although a DAS interrogator represents a significant upfront investment, that cost can be amortized over tens of

kilometers of fiber and across multiple bridges monitored simultaneously, keeping the effective per-channel cost substantially lower than that of dedicated sensor networks (L382–390).

Revised manuscript:
Discussion, L386–394: "Telecom as Sensors leverages existing telecom fibers that eliminate the installation expenses of on-site sensing systems. While a DAS interrogator requires a significant upfront investment, this cost can be amortized over tens of kilometers of fiber and across multiple bridges monitored simultaneously, resulting in a per-channel cost of only a few dollars per meter of fiber, which is substantially lower than that of dedicated sensor networks. Also, unlike traditional sensor networks, telecom fibers require no additional long-term maintenance since they are already maintained to serve communication functions."

Comment:
Please specify the type of interrogator used (e.g., single-pulse or dual-pulse) and briefly discuss whether the results could differ between interrogators.

Response:
We thank the reviewer for this helpful suggestion. We have revised the Methods to identify the interrogator models and installation locations. Specifically, the South Korean bridges were monitored with an AP Sensing N51 interrogator installed at Korea Expressway Corporation offices in Cheongju and Siheung, the Sandhill Road bridge was monitored with an Optasense ODH3 interrogator at the Stanford campus fiber array, and the McKee Road overpass and Yerba Buena Road bridge were monitored with an Optasense Quantx interrogator installed in the San Jose City Hall server room (L478–496).

Different interrogator designs can influence measurement characteristics such as sensitivity, noise floor, and frequency response, so some differences in data quality are possible across systems. A systematic comparison between interrogators, however, is beyond the scope of this study.

Revised manuscript:
Methods, DAS data acquisition, L484–501: "The DAS data were collected by an AP Sensing N51 interrogator from telecom fiber-optic cables installed in 1997 for communication between expressway offices. These cables were housed in air-filled PVC conduits and secured to the bridge deck with metal strap clamps. The conduits were clamped to the side of the box girder for the Miho River bridge (Fig.~\ref{fig:fig3}b), to the side of the concrete slab for the Byeongcheon 2 bridge (Extended Data Fig. 1b), and to the top surface of the concrete slab for the Bakdal 2 bridge (Extended Data Fig. 2b). At the Sandhill Road bridge, DAS data were collected by an Optasense ODH3 interrogator from the Stanford campus fiber array~\cite{10.1785/0220220279}, which consists of a loosely deployed optical cable housed in

an air-filled PVC conduit. The cable extends approximately 45 km, beginning in central Stanford campus and running beneath portions of Palo Alto, Menlo Park, and Redwood City. Within the bridge span, the conduit is mechanically clamped to a steel I-girder. At the Yerba Buena Road bridge and the McKee Road overpass, DAS data were collected by an Optasense Quantx interrogator from a 50-km-long telecom fiber operated by the City of San Jose~\cite{liu2025urban}, with the interrogator installed in the city hall server room."

Comment:
A short discussion on the noise level in the data and on the choice of spatial sampling and gauge length would strengthen the methodological section.

Response:
We thank the reviewer for this helpful suggestion. We have strengthened the Methods discussion of spatial sampling parameters. As summarized in Fig. 2 and Methods L514–519, gauge length and channel spacing vary across sites depending on equipment availability and storage constraints. Gauge length acts as a spatial low-pass filter and suppresses short-wavelength components (L225–229, L532–535). This is reflected in the Results: the Sandhill Road and Yerba Buena Road bridges did not yield reliable guided-wave measurements because their spans and/or gauge lengths limited the observable wavelengths (L230–236), and the long gauge length at Sandhill Road also prevented reliable mode-shape estimation (L352–355).

We have also added a sentence in the DAS data acquisition section discussing the trade-offs associated with gauge length and channel spacing, including their effects on signal-to-noise ratio, spatial resolution, and data volume.

Revised manuscript:
Methods, DAS data acquisition, L519–525: "The DAS configurations vary across sites in gauge length, channel spacing, and sampling frequency, depending on storage and equipment availability (Fig.~\ref{fig:fig2}). Gauge length and channel spacing involve inherent trade-offs: longer gauge lengths generally provide higher signal-to-noise ratios but lower spatial resolution, whereas finer channel spacing improves spatial resolution at the expense of greater data redundancy and storage demand."

Comment:
The impact of the sampling frequency on the analysis could be briefly discussed, especially given the low-frequency content of interest.

Response:
The sampling frequencies used in this study range from 200 Hz to 1000 Hz across the six bridges. As noted in the Methods (L529–535), these rates are more than sufficient for the modal

and quasi-static analyses, while in the guided-wave analysis, the effective upper frequency limit is governed jointly by temporal sampling and gauge length.

Revised manuscript:
Methods, DAS data acquisition, L534–540: “The sampling frequencies used in this study are sufficient to capture modal parameters and quasi-static displacement components and therefore exceed the Nyquist requirement by a wide margin. For guided wave analysis, the sampling frequency defines the upper bound of the observable frequency content. In practice, however, the effective upper frequency limit in DAS is also constrained by the gauge length, which acts as a spatial low-pass filter~\cite{https://doi.org/10.1029/2023JB028033}.”

Comment:
Please justify the choice of strain measurements instead of strain-rate, which is often better suited for low-frequency applications and avoids phase unwrapping issues.

Response:
We appreciate this comment. In this study, the choice between strain and strain-rate output was determined by the interrogator used at each site rather than by a deliberate methodological preference. As described in the Methods (L520–529), the South Korean bridges were recorded in strain rate, whereas the California bridges were recorded in strain. For the South Korean data, strain rate was integrated in time before displacement estimation (L676–677). Both output types produced reliable results in the frequency ranges examined here, as supported by their agreement with the validation sensors.

Comment:
A brief discussion on the implications of removing the first modes for bridge monitoring would be helpful.

Response:
We believe this comment refers to the high-pass filtering at 1 Hz applied during the guided wave retrieval workflow (Methods, L579–585; specifically L581–582), which suppresses quasi-static components dominated by vehicle-induced deformation. This filtering is applied specifically in the ambient noise interferometry pipeline to improve the extraction of coherent guided waves. It does not remove structural modes for modal analysis, which is performed separately using the unfiltered low-frequency DAS data (the FDD analysis targets the 1–5 Hz range where bridge resonances occur). The rationale is that the strong quasi-static vehicle responses would dominate the cross-correlation and obscure the weaker guided wave signals if not filtered out. For bridge monitoring, the low-frequency modes are captured through the separate modal analysis pipeline (Figs. 5, 6, and Extended Data Figs. 5–9).

Comment:
Please clarify whether the chosen integration method may influence the results.

Response:
The double spatial integration of strain to obtain displacement is performed using the trapezoidal rule. This method provides second-order accuracy and is well-suited to the relatively smooth strain distributions observed under vehicle loading. We acknowledge that the choice of integration method can, in principle, influence the results, particularly when the strain field exhibits stronger spatial variability, higher-frequency content, or greater noise levels, because different numerical schemes differ in their sensitivity to discretization error and noise propagation. However, under the conditions considered here, the trapezoidal-rule-based approach performed well and yielded displacement estimates with submillimeter accuracy. A systematic comparison of integration methods is beyond the scope of the present paper and will be addressed in ongoing work.

Comment:
It would be helpful to recall the fundamental assumptions of operational modal analysis under ambient excitation.

Response:
We agree that stating the assumptions explicitly improves the methodological transparency. The FDD method relies on the assumption that the ambient excitation is broadband, stationary, and white noise excitation. Under these conditions, the output PSD matrix can be decomposed via SVD to identify modal parameters. For bridges under traffic loading, the excitation is predominantly from vehicle passages, which provides reasonably broadband forcing. While traffic is not perfectly stationary or spatially white, previous studies have shown that FDD is robust to moderate violations of these assumptions (Liu et al. 2023), particularly when sufficient data duration is available. We have added a brief statement of these assumptions to the Methods section.

Revised manuscript:
Methods, Modal analysis using frequency domain decomposition, L694-700: "This output-only technique is well-suited for analyzing ambient vibrations, as it operates directly on the measured response signals and does not require knowledge of the input excitation. Its standard formulation assumes broadband, approximately stationary, and spatially uncorrelated excitation. Although these assumptions are only approximately satisfied under traffic loading, vehicle-induced excitation is typically sufficiently broadband for practical application."

Comment:

Since spatial resolution depends on gauge length, please clarify that modal shape resolution may differ between bridges.

Response:
The reviewer raises a valid point. The gauge length acts as a spatial averaging window, effectively limiting the spatial resolution of mode-shape estimation. Bridges with shorter gauge lengths can resolve shorter-wavelength mode shapes more reliably than those with longer gauge lengths. The manuscript already notes this effect in the Results (L316–318, L352–355), and we have added an explicit statement in the FDD Methods section (L712–715) clarifying that the achievable mode-shape resolution varies between bridges because gauge length differs across sites.

Revised manuscript:
Methods, Modal analysis using frequency domain decomposition, L716–720: "This dense spatial sampling provides a high-resolution view of the mode shapes along the bridge superstructure. Note that because gauge length varies across sites, the achievable spatial resolution of mode shapes differs between bridges: shorter gauge lengths permit the identification of higher-order modes with shorter spatial wavelengths."

Comment (Remarks on code availability):
To facilitate the reuse of the provided code by the community, I strongly encourage the authors to include a clear and comprehensive README file...

Response:
We fully agree with this recommendation. As noted in our response to Reviewer #1, we have added a comprehensive README file to the code repository.

Response to Reviewer 3:

The novelty of the study should be highlighted better by i) referring to more recent literature (especially for the review papers used, there are much more recent review papers), ii) stressing the efforts to overcome the problems associated with SHM in the last decades (see review paper by Farrar et al., 2025), iii) distinguishing this work from the earlier works of the authors clearly and ensuring that it is not incremental. See for example: Liu et al. (2023), Liu et al. (2025), Donadello et al. (2024).

Response:
We thank Reviewer #3 for this important comment. We agree that the novelty of the study should be better positioned within the recent SHM literature and more clearly distinguished from our earlier work. In response, we revised the Introduction and Discussion to (i) incorporate more recent SHM review papers, including Farrar et al. (2025) and recent bridge-focused reviews, (ii) frame the present study in the context of the long-standing bottlenecks that have limited SHM deployment, and (iii) clarify how part of this manuscript extends Liu et al. (2023) and differs from Liu et al. (2025) and Donadello et al. (2024).

Specifically, following Farrar et al. (2025), we now stress that SHM has advanced by repeatedly addressing stagnation points associated with real-world deployment, including sparse sensing, installation and maintenance cost, environmental and operational variability, and the difficulty of translating laboratory demonstrations into scalable in-situ monitoring. We also note that guided-wave approaches, although highly sensitive to localized damage, have generally remained difficult to deploy for online, in-situ monitoring. We therefore clarify that the novelty of the present study lies not simply in using telecom fiber for modal analysis, but in showing that pre-existing telecom fibers can serve as a scalable bridge-monitoring architecture capable of guided wavefield retrieval, quasi-static displacement estimation, modal analysis, and long-term monitoring on operational bridges.

We have also clarified how the present manuscript extends our earlier studies and related recent literature. Liu et al. (2023) demonstrated the feasibility of modal analysis-based bridge monitoring using telecom fiber on a single continuous three-span bridge, with a focus on resonant frequencies and mode shapes. Liu et al. (2025) established the broader concept of urban sensing using existing fiber-optic networks, but did not address bridge-specific SHM. Donadello et al. (2024) provided important adjacent literature by showing that operational telecommunication links can support seismic monitoring and record traffic-related and infrastructure-resonance signals, but it did not present the bridge-monitoring framework developed here. In contrast, the present study validates Telecom as Sensors across six bridges in two countries and extends beyond prior modal-analysis-focused work by retrieving multi-mode guided wavefields across bridge superstructures, estimating quasi-static displacements with

sub-millimeter validation, and demonstrating year-long monitoring. We revised the manuscript accordingly to make clear that this paper is a substantial extension rather than an incremental update.

Revised manuscript:
Introduction, L74–89: "Recent SHM and bridge-monitoring reviews highlight that, despite these advances, widespread field deployment remains limited by recurring bottlenecks including sparse sensing, installation and maintenance cost, environmental and operational variability, and the difficulty of translating laboratory demonstrations into scalable in-situ systems [12]. Farrar et al. [13] describe these as stagnation points that have shaped the development of SHM over the last decades. They further note that guided-wave approaches, although highly sensitive to localized damage, have generally remained difficult to deploy for online, in-situ monitoring. In bridge monitoring, these challenges are especially acute because resolving localized structural response requires dense spatial sensing, whereas conventional sensing systems remain costly to install and maintain over large infrastructure networks."

Analysis of dynamic responses, L290–296: "DAS using telecom fiber-optic cables coupled to bridges also enables reliable measurement of bridge dynamic responses. Liu et al. [28] first performed modal analysis using Telecom as Sensors on a continuous three-span concrete bridge, successfully estimating resonant frequencies and mode shapes. Recently, Rodet et al. [29] further evaluated the feasibility of using dark fiber to monitor multiple bridges across an extended fiber network. In this study, we extend the validation of Telecom as Sensors for dynamic response monitoring to additional bridges with diverse structural types (box girders and composite concrete–steel I-beams), span configurations, and lengths."

Discussion, L404–412: "Beyond cost efficiency, the high spatiotemporal resolution of DAS provides unique measurement capabilities. It enables meter-scale resolution of bridge mode shapes, demonstrating that telecom fiber networks can be used for modal analysis, and it further enables continuous monitoring of guided wavefields along the bridge superstructure and quasi-static displacement estimation from the same sensing system. This combination of capabilities, together with validation across six bridges and year-long monitoring, distinguishes the present work from earlier telecom-fiber studies."

Comment:
The claims of "maintenance free" and "highly accurate" should be explained further.

Response:
We thank the reviewer for this comment. The term "maintenance-free" (L98-101) refers to the fact that telecom fiber-optic cables are already maintained by telecommunications operators for communication purposes, so no additional maintenance burden falls on the structural monitoring

side. This is in contrast to dedicated sensor networks, which require periodic battery replacement, recalibration, and connector maintenance. We have revised the text to clarify this intended meaning.

Regarding “highly accurate,” this is supported by quantitative results throughout the paper: sub-millimeter displacement accuracy (RMSE of 0.12 mm and 0.08 mm compared to LDV), 100% accuracy in resonant frequency identification for the Miho River bridge, and MAC values of up to 0.94 for mode shapes. We have removed “high accuracy” before giving the evidence in the following paragraph.

Revised manuscript:
Introduction, revised L98–101: “...transforming them into extensive sensing networks that require no additional maintenance beyond what is already provided for communication functions, redefining how and where structural monitoring can occur.”

Comment:
According to my understanding, the time-history of DAS presented in Fig (b), whose magnitude is between 0.4–0.6, seems to differ from the plot presented in Fig (c). For the location of the sensor (b), the spectrum in Fig (c) changes from negative to positive values (blue to red) – different from the values of Fig. (b), even though the location of the peak seems correct. Please explain/revise.

Response:
We appreciate the reviewer’s careful examination of Figure 1. The apparent discrepancy arises from the different normalization used in panels (b) and (c). Panel (b) shows the raw DAS time series at a single channel near mid-span, with the full time-space data normalized to [0, 1]. Panel (c) shows the same event in a space–time representation, where the color scale was normalized separately to the range [-1, 1] for visualization across all channels. As a result, the signal in panel (c) appears to vary from negative to positive values, even though the shape and peak location is consistent with panel (b). We have revised the figure accordingly: panel (c) is now normalized to [-1, 1], and the trace shown in panel (b) is taken directly from the same data displayed in panel (c), so that the correspondence between the two panels is clear.

Revised manuscript:
Updated Figure 1:

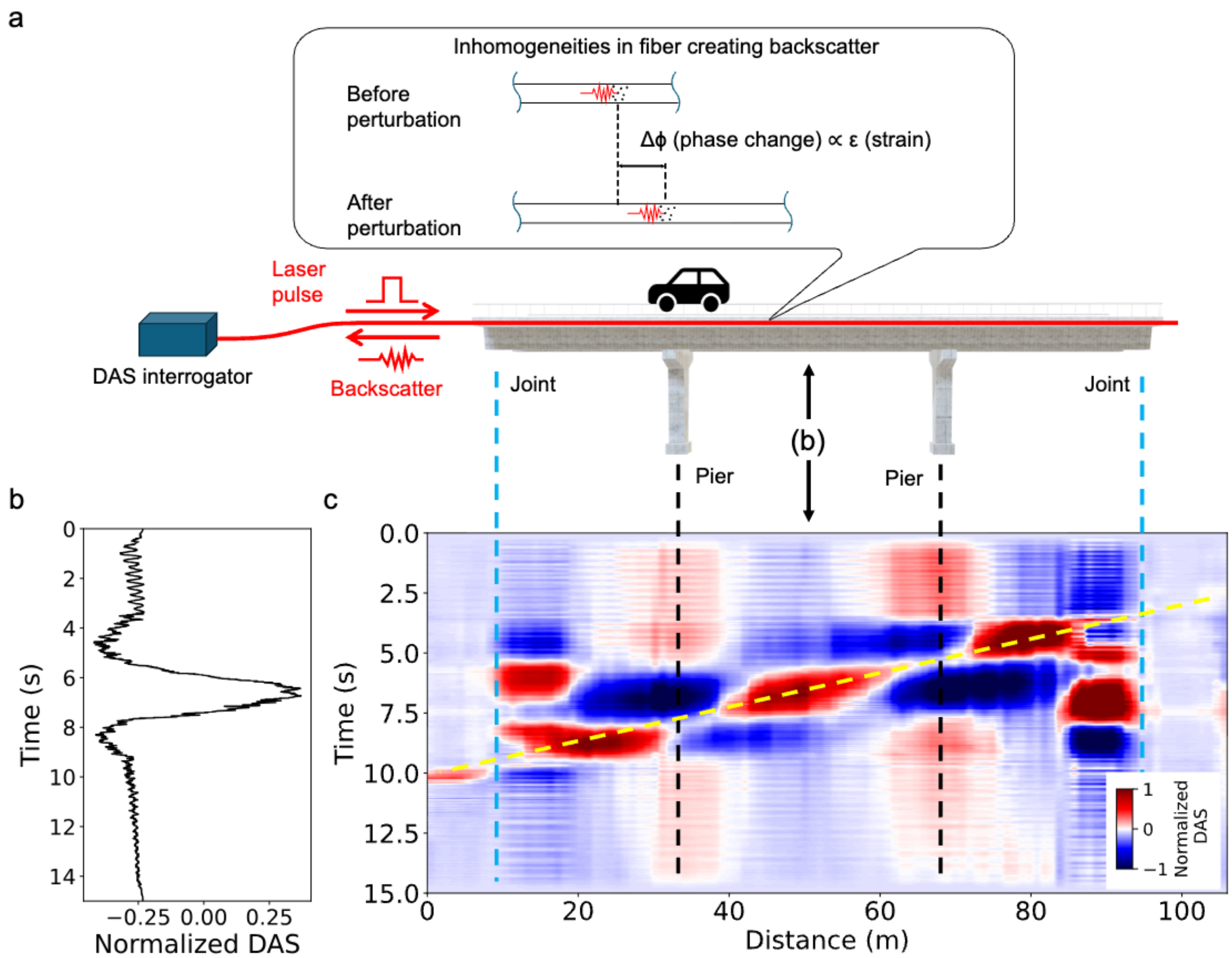

Comment:
It is not clear how the Telecom as Sensors are attached to the bridge structures. Is the same method applied for all of them? What are the implications of the type of the attachment and its location on the bridge?

Response:
The attachment methods are described in the Methods section "DAS data acquisition" (L475–500) and are not the same for all bridges. In all cases, the telecom cables are housed in conduits, but the coupling to the bridge differs:
– Miho River bridge: conduit clamped to the side of the concrete box girder (Fig. 3b).
– Byeongcheon 2 bridge: conduit clamped to the side of the concrete slab (Extended Data Fig. 1b).
– Bakdal 2 bridge: cable laid along the top surface of the concrete slab (Extended Data Fig. 2b).
– Sandhill Road bridge: conduit clamped to a steel I-girder.

– Yerba Buena Road bridge: cable routed along the top surface of the bottom flange of the concrete box girder, based on the design drawings, although current site access did not permit a photograph.
– McKee Road overpass: cable location and coupling method could not be visually confirmed.

To further address this point, we have added a dedicated paragraph in the Methods section discussing coupling mechanisms and their potential effects on measurements. Specifically, the telecom cables are housed in air-filled PVC conduits and secured to the bridge with metal clamps (Figs. 3b, Extended Data Figs. 1b, 2b). This indirect coupling through the conduit introduces mechanical filtering that can attenuate high-frequency signals and reduce strain transfer efficiency compared to directly bonded fibers. The Byeongcheon 2 bridge case (Supplementary Fig. 3) illustrates how inadequate coupling can preclude reliable quasi-static and modal analysis, while the successful results on the remaining five bridges demonstrate that the clamp-based coupling, when reasonably tight, is sufficient for the analyses presented.

Revised manuscript:
Methods, DAS data acquisition, L506–518:
"The quality and interpretability of DAS measurements depend on the mechanical coupling between the telecom cable and the bridge, which governs strain transfer from the structure to the fiber. Because the fiber is coupled through a conduit that is mechanically clamped to the structure, rather than being directly bonded to the bridge, spatial variability in clamp tightness and conduit contact can produce along-cable variations in overall measurement quality. The Byeongcheon 2 bridge illustrates that inadequate coupling (Supplementary Fig. 3) can preclude reliable recovery of quasi-static displacement and degrade modal analysis performance. In contrast, the successful results from the other five bridges indicate that clamp-based coupling, when reasonably tight and continuous, is sufficient for the dynamic response, guided-wavefield, and quasi-static displacement analyses presented here."

Comment:
Figure 3g–h: I do not understand the explanation in the Results section. It is very unlikely that the first bridge modes are at 40–50Hz.

Response:
We thank the reviewer for this comment and agree that the original wording could be misread as referring to the bridge's structural resonance modes. The modes shown in Figs. 3g–h are not global structural resonance modes of the bridge. Rather, they are guided wave cross-sectional modes obtained from the SAFE (Semi-Analytical Finite Element) simulation. Structural resonance modes describe the vibration of the entire bridge as a global structure and, for this bridge, occur at much lower frequencies (approximately 2–4 Hz, as shown in Fig. 5a). In contrast, the modes in Figs. 3g–h describe the local deformation pattern of the bridge

cross-section associated with propagating guided waves at selected frequencies along the dispersion curves. Specifically, Fig. 3g shows the antisymmetric flexural-type A0 guided wave mode at 40 Hz, and Fig. 3h shows the symmetric extensional-type S0 guided wave mode at 50 Hz. These frequencies were selected arbitrarily only to illustrate the characteristic cross-sectional shapes of the two fundamental guided wave modes at representative points on their dispersion branches (marked by the blue and green triangles in Fig. 3f). We have revised the text to make this distinction explicit and to avoid confusion with the structural resonance modes.

Revised manuscript:
Methods, Numerical modeling of guided wave modes, L624–631: "... This method assumes (1) an infinitely long structure, (2) structural uniformity along the z-direction, and (3) harmonic wave propagation, such that the displacement field can be written as $\mathbf{u}(x,y,z,t) = \hat{\mathbf{u}}(x,y)e^{i(kz - \omega t)}$, where $\hat{\mathbf{u}}(x,y)$ is the cross-sectional mode shape. Here, mode shape $\hat{\mathbf{u}}(x,y)$ denotes the guided-wave displacement distribution over the bridge cross-section obtained from the SAFE simulation, rather than a global structural resonance mode shape of the entire bridge."

Comment:
Sandhill Road bridge and Yerba Buena Road bridge examples show the limitations of the proposed method, that are the structural and sensing constraints mentioned. It might be useful to summarize those in the discussion section.

Response:
We agree that summarizing the identified limitations would strengthen the Discussion. In addition to the structural and sensing constraints observed at the Sandhill Road bridge and the Yerba Buena Road bridge, coupling quality also affects the achievable measurements. Specifically, the Sandhill Road bridge is too short (29.3 m) relative to the measurable wavelengths, preventing reliable guided-wave and mode-shape retrieval; the gauge lengths at both bridges (16.3 m for Sandhill Road and 10 m for Yerba Buena Road) are too long, exceeding half of the dominant wavelength and attenuating guided-wave signals; and at the Byeongcheon 2 bridge, poor fiber–bridge coupling degraded the signal quality, precluding reliable quasi-static displacement recovery and weakening modal analysis. These limitations are described in the Results (L230–236, L280–281, and L299–301), and we have now added a summary paragraph in the Discussion (L409–425).

Revised manuscript:
Discussion, L413–429: "Minor discrepancies remain between our results and those obtained from validation sensors or numerical simulations, and several factors, including cable-to-bridge coupling strength, DAS interrogator settings, and environmental noise, influence the reliability of DAS-based measurements. The bridge examples in this study further highlight these practical

limitations: at Sandhill Road, the short span relative to the DAS gauge length prevented reliable guided wavefield and mode shape retrieval, while at Yerba Buena Road the 10~m gauge length similarly constrained guided wave observations. At Byeongcheon 2, poor fiber--bridge coupling degraded the signal quality, precluding reliable quasi-static displacement recovery and weakening modal-analysis performance. Future research should systematically characterize the impact of these factors, particularly coupling conditions and sensing configuration. Ad hoc signal processing and calibration methods could be developed to mitigate their effects~\cite{kennett2025effect,Lindsey2020}. Additionally, DAS generates large volumes of high-rate data, necessitating investment in efficient signal processing pipelines, storage systems, and visualization methods."

Comment:
Do Telecom as Sensors measure in the longitudinal direction of the cables only, in-line with the strains/strain rates measurements or in three orthogonal directions as well? Can you please clarify?

Response:
We thank the reviewer for this important question. DAS measures strain (or strain rate) only along the longitudinal axis of the fiber; it does not directly measure motion in three orthogonal directions. This is a fundamental characteristic of DAS, as the backscattered optical signal is sensitive only to changes in optical path length along the fiber axis, and therefore to axial strain. We have clarified this point in the Introduction (L106–109): "By analyzing the phase shifts between the transmitted and backscattered signals, DAS retrieves axial dynamic strain or strain rate along tens to hundreds of kilometers of fiber with meter-scale resolution and kilohertz sampling frequency."

However, the measured axial strain can result not only from direct longitudinal deformation, but also from flexural deformation of the structure when the fiber is located away from the neutral axis. For this reason, DAS can still be used to infer vertical displacement through double spatial integration of the longitudinal strain.

We also note in the revised manuscript that, in practice, combining telecom-fiber DAS with a limited number of conventional sensors may be beneficial for validation and for capturing response components that are not directly accessible to DAS alone, such as transverse vibrations when the fiber is oriented along the bridge axis.

Revised manuscript:
Discussion, L369–379: "Notably, many of the analyses presented here, including guided wavefield retrieval, resonant frequency identification, and mode shape extraction, are performed directly from the DAS data without requiring numerical models or reference sensors. In this

study, the numerical models and conventional sensors were used exclusively for validation. However, absolute displacement estimation requires a one-time calibration based on either a reference measurement or design drawings, provided that the neutral axis location is known. In practice, combining Telecom as Sensors with a limited number of conventional instruments could further improve validation and interpretation, particularly for capturing response components that are not aligned with the fiber axis."

Comment:
Description of the monitored bridges and Fig 2 seem insufficient in terms of details. Although these bridges and structural features/behavior are integral to the study, the presented information gives the impression that they are complementary. I recommend providing more details and figures (if possible).

Response:
We thank the reviewer for this helpful suggestion. Figure 2 summarizes the bridge locations, aerial views, structural types, span configurations, and DAS acquisition parameters for all six bridges. Additional structural details, including cross-sectional geometries, are provided in Fig. 3c and Extended Data Figs. 1c and 2c, and the material properties used in the analyses are described in the Methods (L637–645). Photographs showing the cable installation and coupling conditions are also included in Fig. 3b, Fig. 4a, and Extended Data Figs. 1b, 2b, and 4a.

For these bridges, more detailed design drawings are not available for publication because they are operational infrastructure and subject to sharing restrictions. We have clarified this point in the revised manuscript and have revised the text to better emphasize that the bridge geometry, structural configuration, and cable coupling conditions are integral to the interpretation of the DAS measurements, rather than supplementary information.

Revised manuscript:
Methods, Description of monitored bridges, L451–460: "This study involves six roadway bridges located in South Korea and the United States. The monitored structures span a wide range of lengths from 29.3 m to 700 m, structural types, materials, and span configurations, enabling broad validation of the proposed sensing system under realistic operating conditions. Data collection durations ranged from several days to a year, depending on site access. Fig.~\ref{fig:fig2} summarizes the structural characteristics, span arrangements, and sensing configurations for all monitored bridges. More detailed design drawings for the monitored bridges are not included because these bridges are operational infrastructure, and the corresponding documents are subject to sharing restrictions."

Comment:

Please provide more information on how telecom optical fibers system function, i.e., operated by whom, where the interrogators are installed, their properties, if the data is publicly available, and more practical data to enable others to use the same methodology.

Response:
We thank the reviewer for this suggestion. Much of this information is provided in the DAS data acquisition section. The monitored telecom fibers were operated by different entities depending on the site: the South Korean fibers were owned and operated by the Korea Expressway Corporation, the Sandhill Road measurements used the publicly available Stanford PubDAS array, and the McKee Road and Yerba Buena Road measurements used city telecom fiber operated through municipal infrastructure in San Jose. The DAS interrogators were installed at Korea Expressway Corporation offices in Cheongju and Siheung for the South Korean bridges, at the Stanford campus for the Sandhill Road bridge, and in the San Jose City Hall server room for the McKee Road and Yerba Buena Road bridges. Detailed information is included in the DAS data acquisition section (Pages 17-18).

We also clarify that the interrogator configurations, including gauge length, channel spacing, and sampling frequency, are summarized in Fig. 2 and described in the DAS data acquisition section. In addition, the processed data used for the figures are publicly available in our repository, and the Sandhill Road raw data are available through the PubDAS dataset (https://pubs.geoscienceworld.org/ssa/srl/article/94/2A/983/619682/PubDAS-A-PUBlic-Distributed-Acoustic-Sensing).

Comment:
What are the practical implications of reaching the SNR convergence after approximately 20 hours? Please explain.

Response:
We thank the reviewer for this question. The approximately 20-hour SNR convergence time reported in Supplementary Fig. 1 and Methods L601–609 indicates that about one day of continuous DAS recording under ambient traffic is sufficient to retrieve stable guided wavefields using noise interferometry. This has an important practical implication: the method is compatible with routine, near-daily monitoring using existing telecom fibers, since the fibers are continuously available and DAS systems can operate unattended. In other words, a reliable baseline or updated guided wavefield can be obtained within a daily acquisition window, enabling repeated tracking of dispersion characteristics and other wave propagation properties over time. Such repeat measurements could support long-term monitoring by detecting temporal changes associated with structural evolution. The required convergence time will also depend on traffic intensity, so bridges with higher traffic volumes may require shorter recording durations.

Revised manuscript:
Discussion, L438–443: "In the longer term, time-lapse monitoring could extend beyond resonant frequency tracking to capture changes in wave propagation properties through successive daily measurements, thereby revealing localized damage. Because stable guided wavefields can be retrieved within twenty hours of ambient traffic recording (Supplementary Fig. 4), the approach is compatible with routine daily monitoring."

Comment;
A figure or a chart explaining the methodology step-by-step would be useful; otherwise, it is difficult to follow for someone like me who is not an expert on guided waves. For example, once the guided wave fields and VSGs are obtained, what can they be used for? Then, dispersion images are obtained from VSGs, and what are they used for?

Response:
We thank the reviewer for this suggestion, which will help make the paper more accessible to a broader audience. We have created a new Supplementary Figure presenting a methodology flowchart that summarizes the three analysis pipelines. The flowchart also indicates which numerical models serve as validation tools versus which steps are purely data-driven.

We also revised the text to better explain the purpose of the intermediate outputs. Specifically, the guided wavefields and VSGs provide spatiotemporal representations of wave propagation that can be used to identify propagation patterns, reflections, scattering, and mode conversions. From the VSGs, we derive dispersion images, which are then used to characterize modal dispersion behavior and infer sensitivity to local changes in material properties, geometry, and boundary conditions. As noted in the manuscript, higher-frequency modes are particularly sensitive to local geometric and structural variations (L218–221), while guided-wave responses more generally are sensitive to local changes in material properties, geometry, and boundary conditions (L159–160).

To further address the reviewer's point, we now state more explicitly in the Discussion (L439–442) that, in an SHM context, localized damage such as cracking, delamination, or section loss would be expected to alter the dispersion characteristics and/or generate additional reflections and mode conversions, which could be detected through repeated measurements over time. A systematic evaluation of this sensitivity for damage diagnosis is part of our ongoing/future work.

Revised manuscript:
A new Supplementary Figure has been added showing the methodology flowchart for the three analysis pipelines.

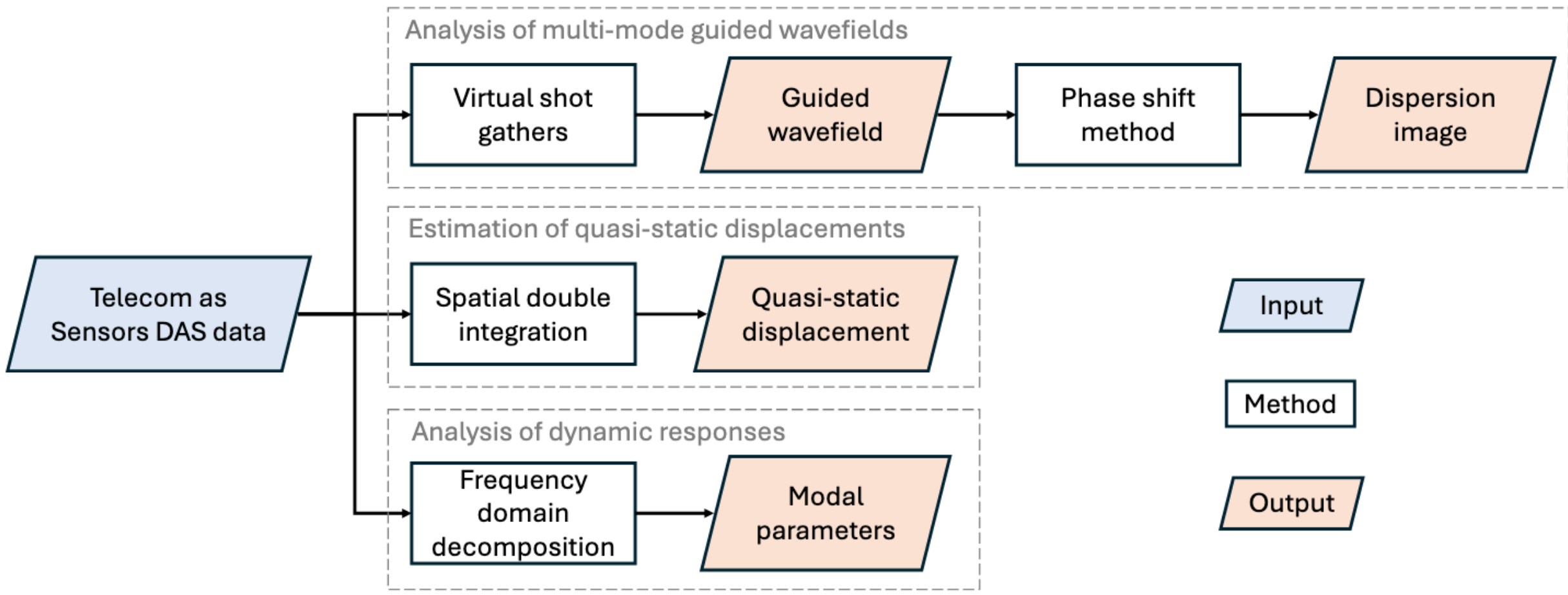

Supplementary Fig. 1: Schematic overview of the analysis framework applied to Telecom as Sensors DAS data for bridge monitoring. Telecom as Sensors DAS data are analysed through three pipelines: guided-wave analysis, quasi-static displacement estimation, and dynamic-response analysis. Guided-wave analysis uses ambient-noise interferometry to construct virtual source gathers (VSGs), from which guided wavefields and dispersion images are derived. Quasi-static displacement is estimated by spatial integration of the DAS strain data. Dynamic responses are analyzed using power spectral density and frequency domain decomposition to identify resonant frequencies and extract mode shapes.

Comment:
The details of the analysis must be provided, i.e., length of the signal, number of FFT used in the transformation, etc. both for the DAS and ACC. It seems that the signal durations for accelerometers are quite short as the resulting spectra are very coarse. If that is the case, why not use longer data? Please explain and justify.

Response:
We thank the reviewer for this comment. We agree that the signal-processing details should be stated more explicitly, and we have revised the manuscript accordingly.

For the FDD and PSD analyses, we used 5 min of data for both DAS and validation sensors, with FFT-based spectral estimates computed from 5 segments using 50% overlap. For the Miho River and Bakdal 2 bridges, the DAS data were low-pass filtered and downsampled to 20 Hz, and the LDV and accelerometer data, originally sampled at 100 Hz, were also downsampled to 20 Hz. For the McKee Road and Yerba Buena Road bridges, the DAS data were low-pass filtered and downsampled from 200 Hz to 50 Hz, while the accelerometer data, originally sampled at 2048 Hz, were downsampled to 64 Hz.

Longer accelerometer records were available at some sites, particularly at McKee Road and Yerba Buena Road. We nevertheless used 5 min windows throughout to maintain a consistent

analysis framework across all bridges and sensing modalities and to remain compatible with the shorter 5–10 min acquisition windows available for the LDV and accelerometer measurements at the South Korean bridges. This choice ensured direct comparability between the DAS and reference-sensor results across all case studies. We have clarified these processing details and the rationale for the selected record lengths in the revised manuscript.

Revised manuscript:
Methods, Modal analysis using frequency domain decomposition, L730–742: "For the FDD and PSD analyses, 5 min of data were used for both DAS and validation sensors, with FFT-based spectral estimates computed from 5 segments using 50\% overlap. For the Miho River and Bakdal 2 bridges, the DAS data were low-pass filtered and downsampled to 20 Hz, and the LDV and accelerometer data, originally sampled at 100 Hz, were downsampled to 20 Hz. For the McKee Road and Yerba Buena Road bridges, the DAS data were low-pass filtered and downsampled from 200 Hz to 50 Hz, while the accelerometer data, originally sampled at 2048 Hz, were downsampled to 64 Hz. The 5 min window was used throughout to maintain a uniform analysis framework across all bridges and sensing modalities and to remain compatible with the shorter 5--10 min LDV and accelerometer acquisition windows, thereby ensuring direct comparability between DAS and reference-sensor results across all case studies."

Comment:
Modal analysis using FDD. How do the modes presented in Figures 3 and 5 compare? Are they related?

Response:
We thank the reviewer for this important question. The modes shown in Figures 3 and 5 are related, but they are not the same type of mode. Figure 3 shows propagating guided wave modes identified from the dispersion images and interpreted using SAFE simulations. In contrast, Figure 5 shows global structural resonance modes extracted from ambient-vibration data using FDD.

The connection between them is that structural resonance can arise from the superposition of left- and right-propagating guided waves, which form standing waves. For a simplified bridge modeled as a Bernoulli–Euler beam, the fundamental resonance occurs when the span length equals half the wavelength, giving the relationship v = 2 f_0 L, where v is wave velocity, f_0 is the resonant frequency, and L is the span length. As shown in Extended Data Fig. 8, the resonance frequencies identified for the Miho River, Bakdal 2, and McKee Road bridges correspond closely to the low-frequency A_0 guided-wave branch observed in the measured dispersion spectra. The standing-wave velocity ranges inferred from the identified resonances agree well with the guided-wave velocities at the same frequencies, indicating that the global

resonance modes in Fig. 5 can be understood as the standing-wave manifestation of the propagating guided waves shown in Fig. 3.

At the same time, we emphasize that the specific modes shown in Figs. 3g–h are not the global resonance modes in Fig. 5. Rather, they are guided-wave cross-sectional modes from the SAFE simulation, illustrating the local cross-sectional deformation patterns of the A_0 and S_0 modes at representative frequencies. We have revised the text to make this distinction explicit and to clarify the physical relationship between guided waves and structural resonance.

Revised manuscript:
Numerical modeling of guided wave modes, L627–630: "... This method assumes (1) an infinitely long structure, (2) structural uniformity along the z-direction, and (3) harmonic wave propagation, such that the displacement field can be written as $\mathbf{u}(x,y,z,t) = \hat{\mathbf{u}}(x,y)e^{i(kz - \omega t)}$, where $\hat{\mathbf{u}}(x,y)$ is the cross-sectional mode shape. Here, mode shape $\hat{\mathbf{u}}(x,y)$ denotes the guided-wave displacement distribution over the bridge cross-section obtained from the SAFE simulation, rather than a global structural resonance mode shape of the entire bridge…"

Comment:
Employed modeling approach with Bernoulli-Euler beams does not permit distinguishing torsional modes or transverse vs. vertical modes. While the Telecom as Sensors pass through one side of the bridge, the same side will show modal displacements for transverse and torsional modes in addition to the vertical modes. How do you distinguish them?

Response:
We thank the reviewer for this important comment. We agree that a simple Bernoulli–Euler beam model and a single DAS sensing line along one side of the bridge do not, by themselves, provide fully unambiguous separation of vertical, transverse, and torsional contributions. In the present study, the numerical models were used primarily to validate the dominant flexural mode shapes observed in the DAS data, and the identified modes are those that show the strongest agreement with the simulations and reference measurements.

Because DAS measures axial strain along the fiber axis, a single fiber line is sensitive to any deformation that produces longitudinal strain at that location. Definitive discrimination among vertical, transverse, and torsional components would therefore require measurements at multiple transverse locations across the bridge deck, or additional independent sensors. We have clarified in the revised manuscript that combining Telecom as Sensors with a limited number of conventional instruments can improve the interpretation of response components that are not aligned with the fiber axis.

Revised manuscript:
Discussion, L376–379: "In practice, combining Telecom as Sensors with a limited number of conventional instruments could further improve validation and interpretation, particularly for capturing response components that are not aligned with the fiber axis."

Point-by-point Response:

Reviewer #1 (Remarks to the Author):

Thank you for addressing the comments. Responses to the other reviewers seem solid as well.

Response: Thank you for your positive assessment!

Reviewer #2 (Remarks to the Author):

Dear Authors,

Thank you for your detailed and thoughtful revision of the manuscript.

I appreciate the considerable effort made to address all reviewers' comments. The revised manuscript is substantially improved, especially regarding the clarification of the scientific novelty, the discussion of practical limitations and coupling conditions, and the additional methodological details provided throughout the paper.

The new explanations concerning guided-wave interpretation, operational constraints, interrogator configurations, and the relationship between modal analysis and guided-wave behavior greatly strengthen the manuscript. The addition of the methodological flowchart and the expanded discussion sections also improve the readability and accessibility of the work.

Overall, this is a very strong, complete, and highly interesting paper for the advancement of DAS-based Structural Health Monitoring. I believe it will be of significant interest to the SHM and fiber-optic sensing communities.

Congratulations on this excellent work.

Best regards

Response: Thank you for your thoughtful and encouraging comments!

Reviewer #3 (Remarks to the Author):

I thank the authors for their diligent work in responding the reviewers' comments. The article has improved significantly and I hope it helps advancing our field and the science.

Response: Thank you for your positive comments! We appreciate your recognition of our revisions.
